# Deep polygenic neural network for predicting and identifying yield-associated genes in Indonesian rice accessions

**DOI:** 10.1038/s41598-022-16075-9

**Published:** 2022-08-15

**Authors:** Nicholas Dominic, Tjeng Wawan Cenggoro, Arif Budiarto, Bens Pardamean

**Affiliations:** 1 BINUS Graduate Program, Bina Nusantara University, Jakarta, 11480 Indonesia; 2 School of Computer Science, Bina Nusantara University, Jakarta, 11480 Indonesia; 3 Bioinformatics and Data Science Research Center, Bina Nusantara University, Jakarta, 11480 Indonesia

**Keywords:** DNA, Computational science, Computer science, Information technology, Scientific data, Statistics, Computer science, Plant genetics

## Abstract

As the fourth most populous country in the world, Indonesia must increase the annual rice production rate to achieve national food security by 2050. One possible solution comes from the nanoscopic level: a genetic variant called Single Nucleotide Polymorphism (SNP), which can express significant yield-associated genes. The prior benchmark of this study utilized a statistical genetics model where no SNP position information and attention mechanism were involved. Hence, we developed a novel deep polygenic neural network, named the NucleoNet model, to address these obstacles. The NucleoNets were constructed with the combination of prominent components that include positional SNP encoding, the context vector, wide models, Elastic Net, and Shannon’s entropy loss. This polygenic modeling obtained up to 2.779 of Mean Squared Error (MSE) with 47.156% of Symmetric Mean Absolute Percentage Error (SMAPE), while revealing 15 new important SNPs. Furthermore, the NucleoNets reduced the MSE score up to 32.28% compared to the Ordinary Least Squares (OLS) model. Through the ablation study, we learned that the combination of Xavier distribution for weights initialization and Normal distribution for biases initialization sparked more various important SNPs throughout 12 chromosomes. Our findings confirmed that the NucleoNet model was successfully outperformed the OLS model and identified important SNPs to Indonesian rice yields.

## Introduction

Yield is one of the superior rice traits which is controlled by multiple genes (called polygenic). Through a Genome-wide Association Study (GWAS), its genetic makeups can be discovered and perceived^1–4^, while still considering any covariates such as climatic conditions^5,6^, field factors^6^, intentional or unintentional environmental damages^7^, and even the dispensable genomes^8^. Rice, as a staple food for over half of the worldwide population, becomes an ideal species model within the monocots plant genomic research community^8,9^ due to its genome’s smallest size (of major cereals), relative simplicity and completeness, dense map, and also ease of manipulation^7,10^. Recall that the Food and Agricultural Organization of the United Nations estimated that by 2050 the worldwide population will increase 32% to 9.1 billion^11^. Particularly, Indonesia had a 1.09% increase in population growth rate by 2020^12,13^ and thus has to increase the annual rice production to feed its entire population and achieve national food security.

GWAS that has been deployed for *indica* and *japonica* subspecies genome sequences database^7,14,15^ in many former studies manifests a remarkable improvement to break the conundrum of identifying what genes influence such traits. By delving deeper to the nanoscopic level, Single Nucleotide Polymorphism (SNP) has been widely applied to predict plant traits^16–23^. In recent years, the yield prediction-related tasks for rice genomic data have been completed using statistical genetic models to machine learning-based open frameworks^24–26^.

Rice yield predictive models should consider confounding variables^27–32^. In Indonesia, a Genetic Generalized Double Pareto Regression (GGDPR)^6^ model incorporates the 1232 Indonesian rice SNPs from 467 accessions with two field indicators and plant varieties as confounding variables. The same dataset is used for this research. GGDPR could control the covariate and allow the repeated measurements for the same rice species in a distinct environment. The algorithm itself, through its shrinkage prior ability, was claimed to successfully handle a condition where the number of the predictors $p$ is greater than the number of samples $n$, $p\gg n$^33,34^, as usually happens in GWAS. With a 0.3% of false discovery rate, GGDPR revealed nine significant SNPs to Indonesian rice yields. One of the SNPs, TBGI050092 (Minor Allele Frequency/MAF = 3%, GGDPR *β* = − 0.186) resides within a gene responsible for rice growth^35,36^. Another intronic SNP, id10003620 (MAF = 5%, GGDPR *β* = 0.515) produces a pentatricopeptide protein, which plays role in stress and developmental response in rice^37^. Meanwhile, the protein product of TBGI272457 (MAF = 12%, GGDPR *β* = − 0.285) equipped rice plants with pathogenic resistance^38,39^. This study uncovers more important SNPs to Indonesian rice yields by constructing a novel deep polygenic neural network model, named the NucleoNets.

In this paper, we present several contributions as follows. First, we designed NucleoNets as the first Artificial Intelligence (AI) based predictive model for the Indonesian rice genomics data. Second, since SNP is scattered in chromosomes with a distinct position index, the learnable SNP positional embedding^40^ was involved in the NucleoNets. Third, we kept covariates (i.e., sample location and variety) in the NucleoNet’s wide model compartement^41^ as proportional memorization against the primary deep model. Fourth, the ablation study was conducted to witness the impact of different parameters initialization against the SNP importance results. Lastly, as the AI-based polygenic modeling for GWAS was completed, we revealed 15 novel important yield-associated SNPs through the NucleoNet’s attention mechanism^42^. Our research offers the availability of the new state-of-the-art with deep learning methods as a stepping-stone to answer the problem of crop yield predictions.

## Methods

### Research workflow

The research problem comprises the development of a deep polygenic neural network to predict Indonesian rice yields and reveal new important yield-associated SNPs. The developed hypothesis is that the Indonesian rice yields prediction performance of the NucleoNet model can outperform the basic linear regression model, i.e. Ordinary Least Squares (OLS) and OLS with an Elastic Net (ENET). To achieve these goals, there are five phases of the methodology.

First, both phenotype and genotype datasets were preprocessed. Second, basic regression modeling was developed to assess the dataset feasibility. Regression is also required for comparison, which is much more commonly used in GWAS. Third, the NucleoNet model was constructed, inspired by the Wide and Deep model. Next, the evaluation phase was done with various metrics to measure the model performance. Lastly, the t-test was conducted to test the hypothesis.

### Data collections

**Figure 1:**
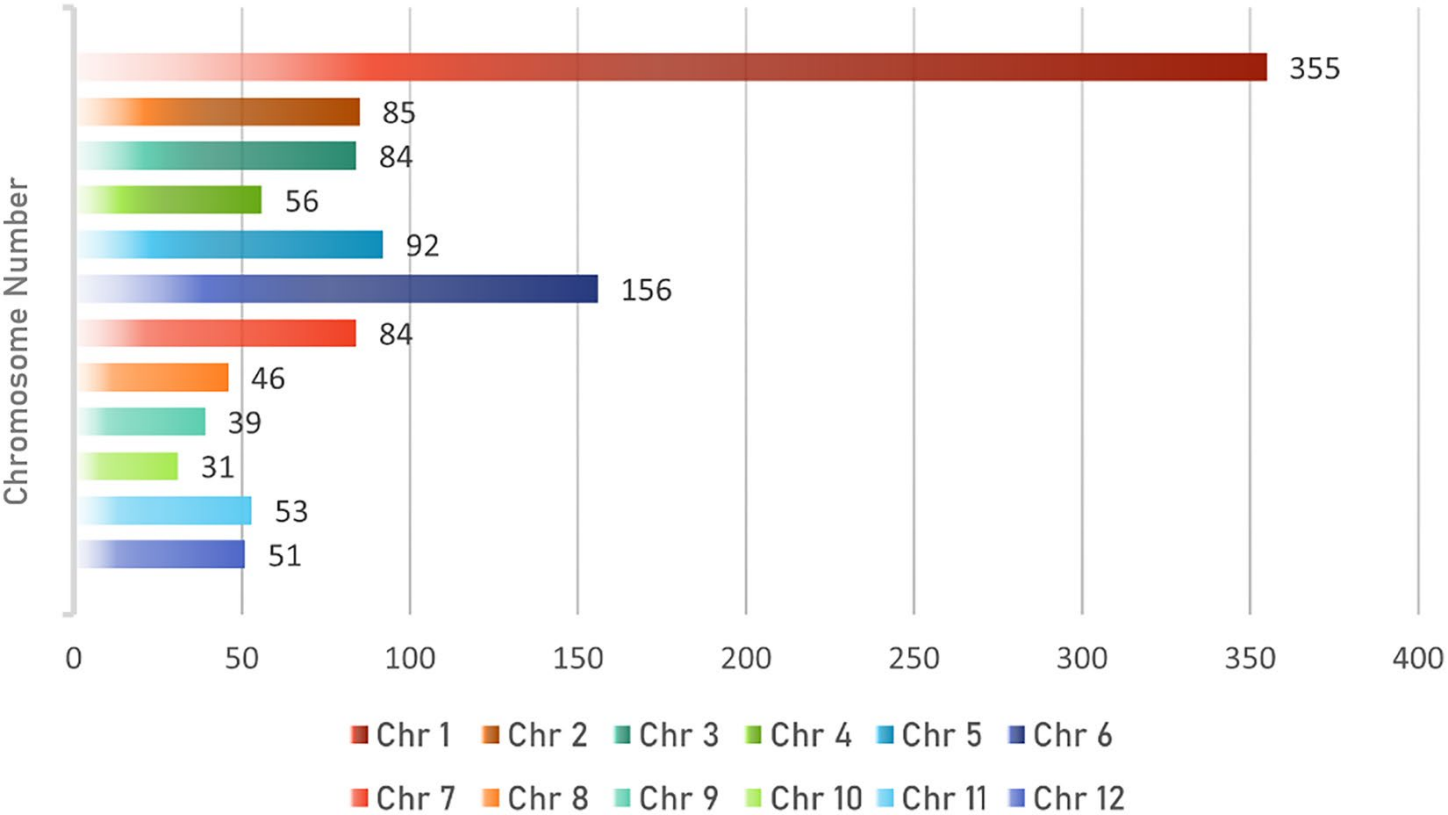
Number of SNPs for each chromosome.

The dataset used for this research was originally curated by the Indonesian Center for Agricultural Biotechnology and Genetic Resources Research and Development (ICABIOGRAD). The database collection consists of 467 rice germplasm samples, 467 × 1536 genotypes (SNPs), and 467 × 4 locations × 12 phenotypes. In detail, the germplasm sample consists of 136 local varieties, 162 improved lines, 11 wild species, 34 near-isogenic lines, 29 released varieties, and 95 newly identified varieties. These samples contain 77 Japonica, 108 Tropical Japonica, and 249 Indica subspecies, leaving the remaining 33 samples with unlabelled subspecies. The Indonesian rice genome consists of 12 chromosomes, which each has different numbers of SNP. The proportion is depicted in Fig. 1. Both sample and phenotype data are in Comma-separated Values (CSV) format files, while genotype data is provided in CSV and PLINK format files.

The basic attributes in the genotype file are chromosome number (*chr*), SNP ID (*snp*), SNP position in DNA sequence (*pos*), reference allele (*ref*), alternative or mutated allele (*alt*), and genotype data/SNP (*gt*) itself. Meanwhile, the phenotype file describes 12 available rice traits (see Table 1 in the Supplementary Information). The rice planting location includes Subang, Citayam, Kuningan, and Greenhouse (a controlled environment). The incomplete rainy season climatic data such as temperature, humidity, wind speed, precipitation, and irradiance were excluded. The other exclusion reason is that the climatic data was reported to be practically identical throughout the locations^6,43^.

### SNP validation

We validated our Indonesian rice SNPs data to the 18,128,777 Rice Genome Project (RGP) and found that only 57 Indonesian rice SNPs (4.63%) were registered in the International Rice Research Institute (IRRI) database (see Table 2 in the Supplementary Information).

### Data preprocessing

This preprocessing phase aims to create a Genotype–Phenotype (GP) table consisting of the following columns: sample ID, sample name, sample location, sample variety, SNP, SNP position, and yield. Note that samples from the Greenhouse were excluded since all yields are unreported (thus, the total sample location is $l$ = 3).

The previous work^6^ reported that the raw genotype data consists of 1536 SNPs with approximately 389 megabases. After the genotype dosage imputation by the Bayesian Imputation Based Association Mapping (BIMBAM) software for SNPs with call rate beyond 25% and removal of monomorphic SNP, 697 rice samples × 1232 SNPs were obtained. The alternative imputation services are Online Plant-ImputeDB or Rice Imputation Server^44^ which utilized cloud computational offloading technology^45^. Note that before the imputation, referring to the raw data we received, the call rate of 9 significant SNPs is 0.222% for TBGI036687, 1.774% for TBGI050092, 0.665% for id4009920, 1.109% for id5014338, 1.330% for both TBGI272457 and id8000244, 20.843% for id7002427, 2.217% for id10003620, and 0% for id12006560. The call rate is calculated by dividing the number of samples that have a null value in their related SNP by the total number of samples.

**Figure 2:**
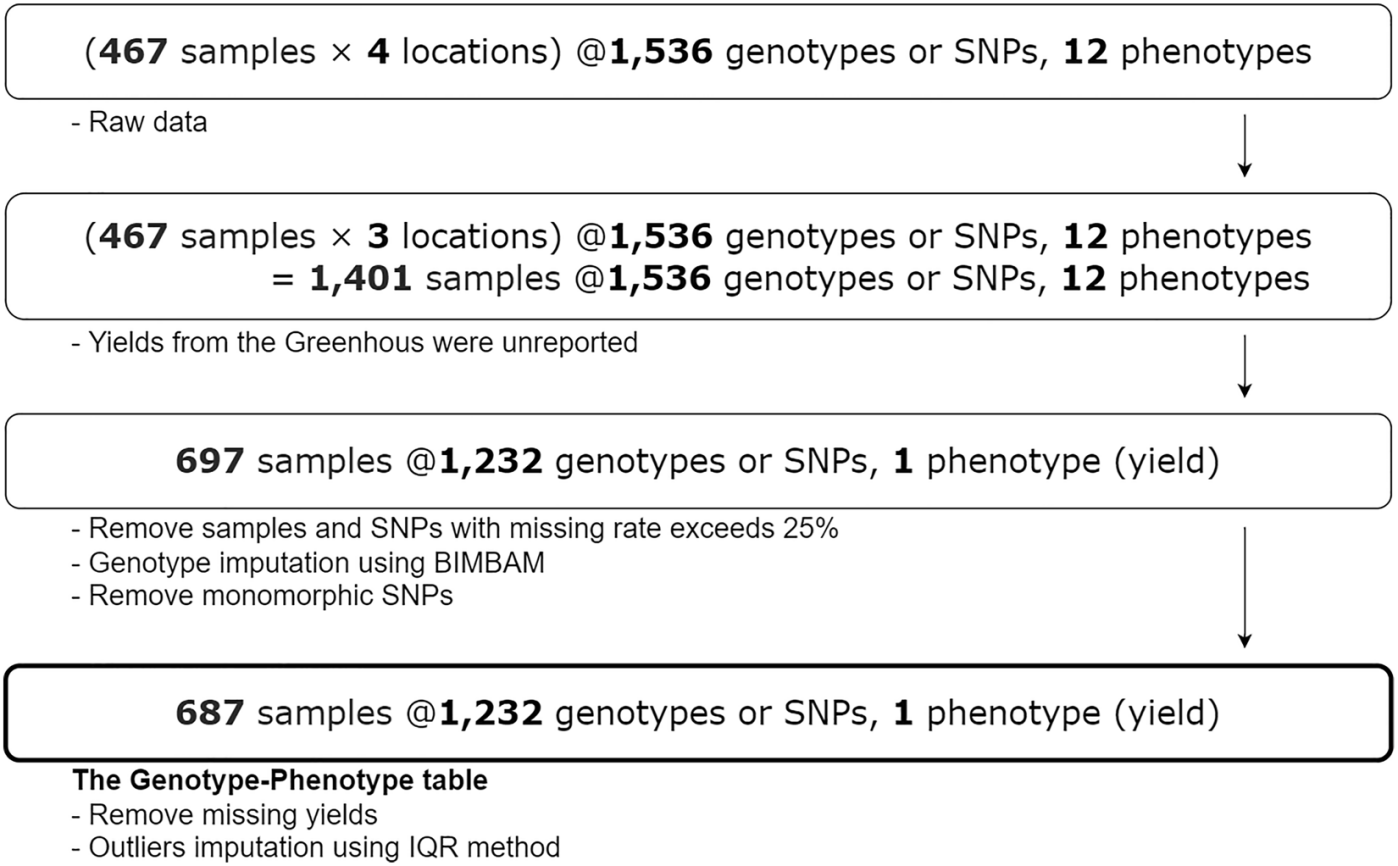
Data preprocessing step.

Next, from the 697 samples, mild and extreme outliers in the yield data were detected by using the Interquartile Range (IQR) method. From here 10 missing yields were dropped and the outliers were imputed with the global mean. Therefore, the final Genotype–Phenotype table has 687 rice samples, with each has 1232 SNPs (genotypes) and 1 yield rate (phenotype to predict). See Fig. 2 for details.

Note that in the genotype dataset, all SNPs were encoded based on the additive model^46^. The scheme encodes SNP according to the total of its alternative allele, as it represents a mutation in one locus (see Table 3 in the Supplementary Information). Genotype dosage, which is implanted within the BIMBAM tool, is a linear transformation technique used to fill the missing genotypes in SNP. It is based on the posterior genotype probabilities^47,48^. Most of the imputed SNPs are in real numbers. To adapt them with the SNP encodings, all real numbers were half-rounded to even (also known as a Banker’s rounding behavior, as applied in Python 3.x).

### Regression modeling

The GP Table data frame was shuffled and 85% of the total data was then reserved for train data. After this splitting, the t rain data has a coefficient of variation (CV) of 1.878, and the test data has a CV of 1.798, which still showed the fair dispersion of yield data. In this regression section, we rendered three experiments. First, all SNPs were included in the Ordinary Least Squares (OLS) as a part of polygenic modeling (Experiment 1). Second, each SNP was regressed to yield as a part of an independent association test or marginal regression (Experiment 2), as commonly found when dealing with GWAS. Third, the Elastic Net (ENET) regression was conducted to see the results under the coefficients penalty (Experiment 3). All SNPs were included when the ENET was performed. Its results were plotted into the correlation heatmap to scrutinize the effects of the alpha constant (used to multiply the penalty term) and L1 ratio tuning. This ratio works by 0 < L1 ratio < 1. Both alpha and L1 ratio spaces follow the arithmetic sequence of ${\mathcal{a}}_{n}={\mathcal{a}}_{0}+nd$, where $n=19$ and ${\mathcal{a}}_{0}=d=0.05$. All significant SNPs from Experiment 1, Experiment 2, and previous research^6^ were gathered and compared. These SNPs were then retrained in the OLS model to seek the best prediction score against the rice yield. The trial was also intended to meticulously examine whether there are beneficial insights and impacts of using only the partial SNP data.

### The NucleoNet modeling

The GP table was loaded and shuffled. A tensor object was then created for SNP data (${x}_{1}$), SNP position data (${x}_{2}$), sample location data (${x}_{3}$), sample variety data (${x}_{4}$), and yield data ($y$). The complete dataset has a format: [[tensor (${x}_{1}$), tensor (${x}_{2}$), tensor (${x}_{3}$), tensor (${x}_{4}$)], tensor ($y$)]. We split the dataset into 70% of training data, 15% of validation data, and 15% of testing data. The fivefold cross-validation was conducted using the training and validation data. We utilized the Hyperopt library which has a Tree-structured Parzen Estimator (TPE) algorithm^49^. Given a search space, Hyperopt returned the best hyperparameters for the model, and hence the validation accuracy can be optimal^50^.

**Figure 3:**
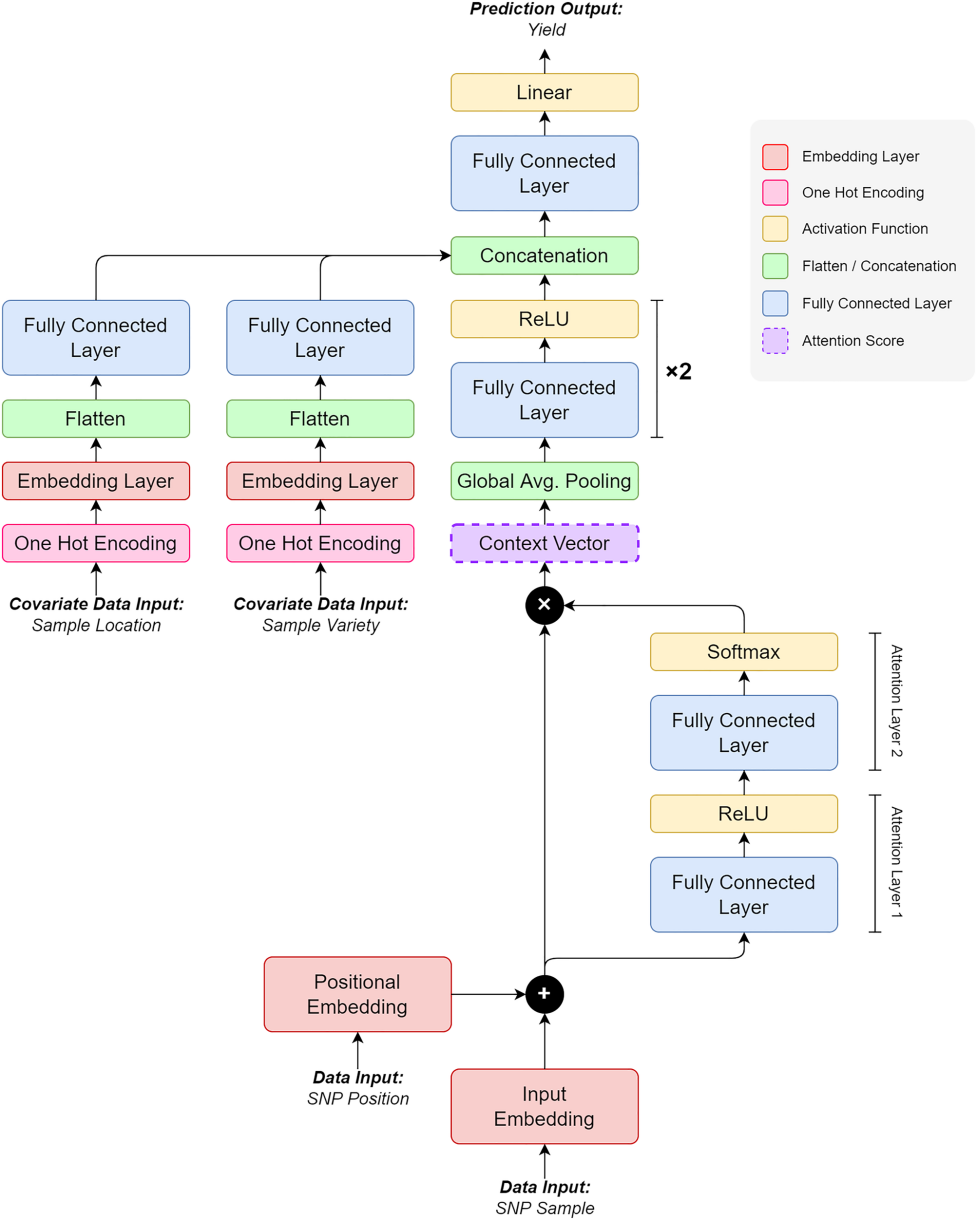
The NucleoNet model.

The design of the NucleoNet model is depicted in Fig. 3. Generally, it consists of a deep model which starts from SNP sample data (${x}_{1}$) and SNP position data (${x}_{2}$) inputs, and a wide model which starts from covariate data (${x}_{3}$ and ${x}_{4}$) inputs. In the deep model, embedding results from both ${x}_{1}$ and ${x}_{2}$ were added up; we called it $x {^{\prime}}$. This $x {^{\prime}}$ was then fed into the attention layers before the attention score (context vector) was obtained. The context vector ${c}_{i}$ acts as an encoder map to the SNP input sequence, formulated as

1 $${c}_{i}={\alpha }_{i}{x}_{i}^{^{\prime}}.$$

${\alpha }_{i}$ is the alignment model as a multi-layer neural network with Softmax activation function (from attention layers). The probability of ${\alpha }_{i}$ reflects the importance of ${x}_{i}^{^{\prime}}$, thus it will be used as a measure of the SNP feature importance. While ${\alpha }_{i}$ was retrieved in the testing stage, the context vector result was passed to the next layer, i.e., Global Average Pooling (GAP), in the training stage. GAP was used to reduce the spatial dimension of the Tensor data with less parameters. Outputs from GAP were then fed to the fully connected layers (FC1 and FC2). The output from FC2 marked the final result from the deep model.

Both covariates were encoded using a one-hot vector before being fed to the embedding layer. The one-hot vector size for the sample location data input (${x}_{3}$) is $l$ = 3, while for the sample variety data input (${x}_{4}$) is $v$ = 467. The flattened output from each layer was then concatenated with FC2 to form the Wide and Deep model. The fully connected layer (FC3) with linear activation function was added in the final layer and hence the NucleoNet model was completed. The prominent NucleoNet compartments are listed in Table 1. Meanwhile, Table 2 describes the detailed Tensor size of each layer in the model. Notice that the final output from Wide Model 1 and Wide Model 2 was reduced to suppress the effect of the covariate against the primary deep model.

**Table 1:** The prominent parts of the NucleoNet model.

No.	Component in model	Purpose
1	Positional encoding^40^	Add SNP position information to the primary SNP data
2	The context vector^42^	As the attention mechanism, to emit the SNP importance value
3	Wide model^41^	Accommodate all covariates
4	Elastic net^51–53^	Penalize all parameters in all layers
5	Entropy loss^54,55^	Control the distribution of attention scores across all SNPs

**Table 2:** Tensor size for each layer in the NucleoNets. In this table, $b$ indicates the batch size, $s$ indicates the length of SNP, $e$ indicates the embedding size, ${a}_{h}$ indicates the number of attention hidden layers, $l$ indicates the number of sample locations, $v$ indicates the number of sample varieties, ${m}_{hd}$ means the MLP hidden layer of the deep model, ${m}_{hw}$ means the MLP hidden layer of the wide model, and FC means the Fully Connected layer.

Deep model	Size	Wide model	Size	Wide deep model	Size
SNP data input (${x}_{1}$)	$[b,s]$	Sample location data input (${x}_{3}$)	$[b, 1]$	Concat	$[b, {o}_{1}+{o}_{2} +{o}_{3}]$
SNP data embedding	$[b,s,e]$	Sample location one hot encoding	$[b, l]$	FC3	$[b, 1]$
SNP position input (${x}_{2}$)	$[b,s]$	Sample location embedding	$[b, l, e]$	Output ($y$)	$[b]$
SNP position embedding	$[b,s,e]$	Sample location flatten	$[b, le]$		
SNP data + position ($x {^{\prime}}$)	$[b,s, e]$	Wide model 1 (${m}_{hw1}/8={o}_{2}$)	$[b, {m}_{hw1}/8]$		
Attention layer 1 (${a}_{1}$)	$[b,s,{a}_{h}]$	Sample variety data input (${x}_{4}$)	$[b, 1]$		
Attention layer 2 (${a}_{2}$)	$[b,s, 1]$	Sample variety one hot encoding	$[b, v]$		
Context vector (${x}^{^{\prime}}{a}_{2}$)	$[b,s,e]$	Sample variety embedding	$[b, v, e]$		
Concatenation (GAP)	$[b,s, 1]$	Sample variety flatten	$[b, ve]$		
FC1	$[b,{m}_{hd1}]$	Wide model 2 (${m}_{hw2}/8={o}_{3}$)	$[b, {m}_{hw2}/8]$		
FC2 (${m}_{hd2}={{\varvec{o}}}_{1}$)	$[b,{m}_{hd2}]$				

We designed three experiments. Experiment 1 is the NucleoNet model with Mean Squared Error (MSE) loss function (called NucleoNetV1). Experiment 2 is the same except there is an additional modified ENET penalty in the loss function (called NucleoNetV2). Note that both ENET and Generalized Double Pareto (GDP) which was implemented in previous research^6^ have the same role in coefficients shrinkage^33,34^. The selection of ENET as shrinkage prior was due to simpler implementation and more commonly used in genomics studies to solve $p\gg n$ problems, such as selection method to eliminate trivial genes^53^, dense SNPs pre-selection^56^, genomic estimated breeding value (GEBV) prediction^57^, pharmacogenetics^58^, and even the epistasis analysis^59^. Equation (1) describes one of the ENET conventions which are used for the glmnet package in R and Scikit-learn in Python^51,52^, overriding the original naïve ENET. The advent of $\frac{1}{2}$ in Eq. (1) is considered to cancel the exponent 2 (from ${\beta }^{2}$) after derivative. For the NucleoNet, which is not a generalized linear model, this modified ENET is more suitable. The term ${w}_{r}$ implies the regularization weight to control this penalty against MSE loss, while $\beta$ denotes the coefficients and $\alpha$ denotes the penalty term. The convex combination is no longer used, so ${\alpha }_{1}+{\alpha }_{2}\ne 1$.

2 $${\widehat{\beta }}_{enet}=MSE+{w}_{r}\left({\alpha }_{1}\left|\beta \right|+\frac{1}{2}{\alpha }_{2}{\beta }^{2}\right);\alpha =\frac{{\lambda }_{1}}{{\lambda }_{1}+{2\lambda }_{2}},$$

3 $$H=-{w}_{H}\sum_{{x}^{^{\prime}}}{p}_{{x}^{^{\prime}}}{\mathrm{log}}_{2}{p}_{{x}^{^{\prime}}}.$$

Experiment 3 is the same as Experiment 2 except there is another additional Shannon’s entropy value^54,55^ in the loss function (called NucleoNetV3). This entropy acts as a control for the dispersion of attention scores across all SNPs. In other words, we prevent the attention score from collapsing to only one SNP. Equation (2) shows the Shannon’s entropy formula used in Experiment 3, where $H$ denotes the Shannon’s entropy value, ${p}_{x {^{\prime}}}$ denotes the probability value of $x {^{\prime}}$, and ${w}_{H}$ denotes the entropy weight to control $H$ against the $MSE$ loss.

Hyperopt was executed for each designed experiment. Due to limited computational resources, Hyperopt parameters were set to 20 of training epoch, 10 of maximum evaluation, and 43 of initial seed. All the best hyperparameters found were retrieved and used for the NucleoNet model mini-batch training in 1000 epochs. We also set 15 as a number of patience, which is a maximum epoch number of tolerance when there is no further improvement in the training.

### Ablation study

Seven ablation studies (ABSTs) in terms of weight initialization were also conducted, as summarized in Table 4 in the Supplementary Information. In the first attempt (ABST-1), we let weights and biases initialization by default in PyTorch, i.e., within the Kaiming Uniform distribution. For all ABSTs, weights and biases in the SNP data embedding, SNP position data embedding, sample location data embedding, sample variety data embedding, and fully connected layer in the deep model were initialized within the $\mathcal{N}(\mu ,{\sigma }^{2})$, which denotes the Normal distribution. In contrast, $\mathcal{U}(a, b)$ denotes the Uniform distribution, as used in ABST-5. From ABST-2 to ABST-7, we modified weights and biases initialization in the attention layer to examine the variability in the SNP importance measures.

Inspired from the previous study^6^ where it was considered $\sigma =\left\{0.5, 1.0, 2.0\right\}$, we also tried to varied the $\sigma$ within the Normal and Uniform distribution. The Xavier Initialization is used to determine ${\sigma }^{2}$ in the Normal distribution by taking ${g}_{r}=\sqrt{2}$ as the gain value for the linear layer with the ReLU activation function. Meanwhile, the Kaiming Initialization is used to determine the lower and upper bound in the Uniform distribution by taking ${g}_{l}=1$ as the gain value for the linear layer. To your preference, ${f}_{i}$ and ${f}_{o}$ in Table 4 in the Supplementary Information means the number of the input and output nodes, respectively.

### Evaluation metrics

Due to the prediction task, the best possible way to measure the model performance on the test dataset is by using $MSE$ or L2 Loss, Root MSE ($RMSE$), Mean Bias Error ($MBE$), Mean Absolute Error ($MAE$) or L1 Loss, Mean Squared Logarithmic Error ($MSLE$), and Symmetric Mean Absolute Percentage Error ($SMAPE$). These metrics are currently the most widely used in the agroindustry field, especially for yield forecasting with machine learning approaches^60,61^. See the Supplementary Information about the selection reason for these metrics. Note that due to the nonlinearity of the dataset, the Coefficient of Determination or R-squared (${R}^{2}$) is unsuitable for the evaluation measurement^32,62^. The $RMSE$, $MBE$, and $MAE$ inequality are defined as $MBE\le MAE\le RMSE\le \sqrt{n}MAE$^63^. A total of 104 testing data were used in both regression and deep learning approaches. The prediction evaluation is based on all these metrics. In addition, the paired t-test (or dependent t-test) was performed for hypothesis testing.

### Hardware, software, and libraries

The research was executed in hardware with specifications of Intel^®^ CoreTM i5-8250U @1.60 GHz (8 CPUs) ~ 1.8 GHz processor, X442UQR/X442UQR.308 system model, 16,384 MB RAM, and Windows 10 (64-bit) operating system. Developer software includes Jupyter Notebook 6.0.1, Rstudio 1.1.463, Preferred Installer Program/PIP 21.2.4, and PLINK 1.9. The main programming language is Python 3.7.1. Python libraries used are Torch 1.9.0, Pandas 1.3.3, Scikit-allel 1.3.5, Scikit-learn 0.24.2, Hyperopt 0.2.5, Statsmodels 0.12.2, Statistics 1.0.3.5, Matplotlib 3.4.3, Seaborn 0.11.2, and Numpy 1.19.5. All libraries may have the alternative and can be installed through the Python package manager (i.e., PIP).

## Results

### Statistical analysis

The same 467 species were grown in three distinct locations, i.e., Kuningan (2010–2011), Subang (2011–2012), and Citayam (2012–2013). Referred from the previous research^6^, the total data used is 697 samples. All 10 missing yields from Citayam were dropped, leaving 687 samples. The outliers were detected using the Interquartile Range (IQR) method, with Lower Outer Fence (LOF) of − 6.38, Lower Inner Fence (LIF) of − 2.19, Upper Inner Fence (UIF) of 8.98, and Upper Outer Fence (UOF) of 13.17. Precisely, 27 mild outliers were appeared and then imputed by 3.449 as the global mean of rice yield. No extreme outlier was found.

As we plotted the density distribution of rice yields in each location, 150 samples from Kuningan (5.01 ± 1.98) has the Skewness coefficient ${\gamma }_{1}$ of 0.14 and the Kurtosis coefficient ${\gamma }_{2}$ of − 0.86, 124 samples from Subang (3.62 ± 1.82) has ${\gamma }_{1}$ of 0.08 and ${\gamma }_{2}$ of − 0.85, and 413 samples from Citayam (2.83 ± 1.43) has ${\gamma }_{1}$ of 0.19 and ${\gamma }_{2}$ of − 0.61. Samples in Citayam have the largest ${\gamma }_{1}$, which means mostly the yield $\le \mu$. However, the samples in Kuningan and Subang have the lowest ${\gamma }_{2}$, which means the yield is more varied than the rest. Higher $\sigma$ from both supports the statement. Overall, all 687 data (3.44 ± 1.85, ${\gamma }_{1}$ = 0.53, ${\gamma }_{2}$ = − 0.06) is close to the normal distribution (since ${\gamma }_{2}\approx 0$), but still positively skewed (since ${\gamma }_{1}>0$). See the distribution histograms in Table 5 in the Supplementary Information.

### Ordinary least squares results

From the OLS, which is part of Experiment 1, we obtained 16 significant SNPs. From Experiment 2, where we regressed each SNP to yield, we obtained 36 significant SNPs. See the results in Table 3. All significant SNPs found in Experiment 1, Experiment 2, and previous research were once again regressed with the normal OLS and OLS + ENET models. Unfortunately, it seems that there is no prominent result by using only the partial SNP data. Nevertheless, the OLS + ENET model still outperformed the normal OLS results. Compare them in Tables 6 and 7 in the Supplementary Information. To these findings, we chose to utilize all SNPs in the deep learning model training instead. In Experiment 3, we conducted a simulation to scrutinize the effects of alpha constant (used to multiply the penalty term) and L1 ratio tuning in the ENET. Throughout these simulations, we can perceive that the L2 penalty domineeringly affects the outcome. To grasp the full impact of this ENET hyperparameter configuration in six different prediction measures, please refer to Fig. 2 in Supplementary Information. This trial consumed about 30 min 40 s of execution time (ET).

**Table 3:** NucleoNets model comparison with other models. ✓: This symbol means the related part is available in the model. ✖: This symbol means the related part is unavailable in the model. *Not mentioned in the original paper^6^. **The Scikit-learn library does not support the *p*-value calculation. On the contrary, the Stasmodels library does not have an ENET function. ***NucleoNets results from ABST-6.

Polygenic model	GGDPR	OLS	OLS + ENET	NucleoNetV1	NucleoNetV2	NucleoNetV3	Wide and deep model
Total Indonesian rice SNPs	1232	1232	1232	1232	1232	1232	1232
SNP data	✓	✓	✓	✓	✓	✓	✓
SNP position data	✖	✖	✖	✖	✖	✖	✓
Covariate: sample location	✓	✓	✓	✓	✓	✓	✓
Covariate: sample variety	✓	✓	✓	✓	✓	✓	✓
Shrinkage prior/regularization	Generalized double pareto	✖	ENET	✖	Modified ENET	Modified ENET	Modified ENET
Shannon’s entropy	✖	✖	✖	✖	✖	✓	✓
Evaluation: MSE	N/A*	4.104	2.517	2.779***	2.799***	2.863***	8.535
Evaluation: RMSE	N/A*	2.026	1.587	1.667	1.673	1.692	2.921
Evaluation: MBE	N/A*	− 0.236	− 0.404	0.099	0.015	− 0.074	− 2.148
Evaluation: MAE	N/A*	1.673	1.321	1.407	1.412	1.433	2.497
Evaluation: MSLE	N/A*	0.286	0.185	0.184	0.191	0.197	0.468
Evaluation: SMAPE	N/A*	64.843%	45.432%	47.156%	47.960%	47.481%	63.668%
Significance/importance level	N/A*	$p<0.05$	N/A**	$a {^{\prime}}\ge 0.025$	$a {^{\prime}}\ge 0.025$	$a {^{\prime}}\ge 0.025$	N/A
Number of significant/important SNP	9	16	N/A**	29	35	23	N/A
Execution time	N/A*	< 2 s	< 2 s	1630 s	5120 s	4910 s	6070 s

### The NucleoNets results

**Figure 4:**
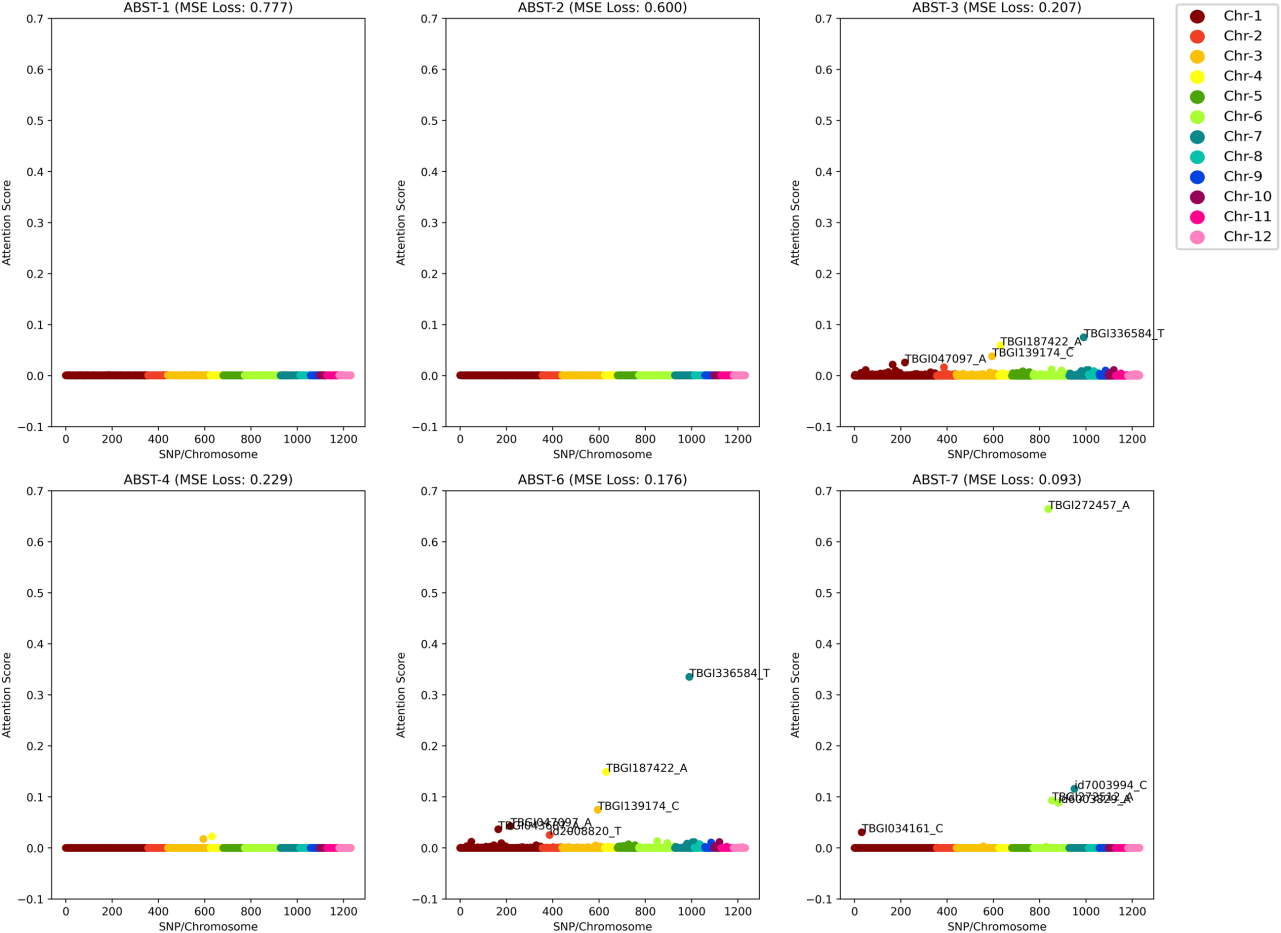
Ablation study results testing for one random sample.

**Figure 5:**
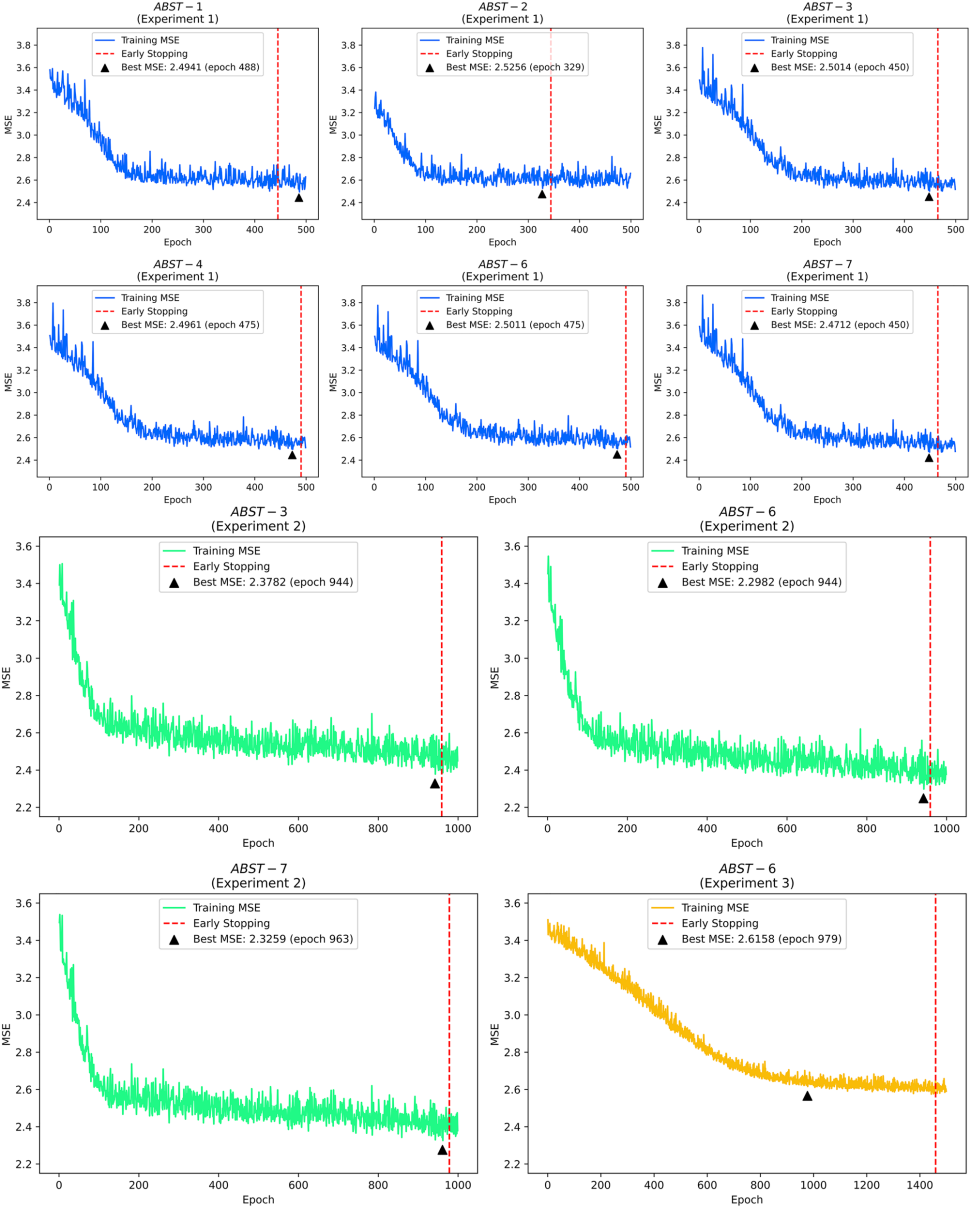
The NucleoNets training plots.

In Experiment 1, we performed 7 ablation studies (ABSTs) with distinct weights and biases initialization. Each of the ABSTs used hyperparameters found by Hyperopt, as inscribes in Table 8 in the Supplementary Information. This validation scheme gave an MSE of 3.032 and consumed about 1 h of ET. In contrast, the training time took approximately 1600 s for 500 epochs. As we can scrutinize in Table 9—Experiment 1 (Supplementary Information), there is only a slightly different result between each ABST. Referring to the MSE measurement, NucleoNetV1 gave testing scores of 2.890, 2.843, 2.785, 2.813, 2.779, and 2.794 for ABST-1, ABST-2, ABST-3, ABST-4, ABST-6, and ABST-7, respectively. The key to interpreting these results resided in their Manhattan plot, as depicted in Fig. 4. Note that for all plots, we utilized the same one random sample for uniform comparison. Since we discovered that ABST-3, ABST-6, and ABST-7 sparked more various important SNPs, the mixed-use of Xavier Initialization in attention layers was maintained throughout the rest of the experiments. All training plots for NucleoNetV1 are diagrammed in Fig. 5 (marked in blue).

Experiment 2 was run in 1000 epochs with approximately 5000 s of ET. The validation scheme for NucleoNetV2 obtained an MSE of 3.097 and consumed about 1 h 16 min of ET. Referring to the MSE measurement, NucleoNetV2 gave testing scores of 2.782, 2.799, and 3.035 for ABST-3, ABST-6, and ABST-7. See Table 9—Experiment 2 (Supplementary Information) for results from other metrics. In ABST-3, both attention layers used Xavier Normal distribution to initiate weights and biases. Meanwhile, in ABST-6, the Xavier Normal distribution was initialized in the first attention layer and in ABST-7 the same distribution was initialized in the second attention layer. Training plots for NucleoNetV2 are diagrammed in Fig. 5 (marked in green).

In Experiment 3, we only reported the NucleoNetV3 testing results on ABST-6 since the SNP importance occurrence variation in the Manhattan plot is much higher than ABST-3 or ABST-7. The validation scheme for NucleoNetV3 obtained an MSE of 3.233 and consumed about 1 h 35 min of ET. NucleoNetV3 gave an MSE of 2.863, trained within 1,000 epochs and consumed approximately 4900 s of ET. See Table 9—Experiment 3 (Supplementary Information) for results from other metrics. For uniformity purposes in all NucleoNets, we determined the result from ABST-6 as primary and therefore are used as comparisons with other models. Training plots for NucleoNetV2 and NucleoNetV3 are diagrammed in Fig. 5 (marked in gold).

In addition, to compare with other deep neural network model and to show the advantage of the NucleoNets, wide and deep model was trained with the same hyperparameters setting of NucleoNetV3. As shown in Table 3, the absence of an attention mechanism reduced the performance. Hence, it is proved that NucleoNets not only obtained superior testing results by using the attention layer but also can emit important SNPs to rice yield.

**Figure 6:**
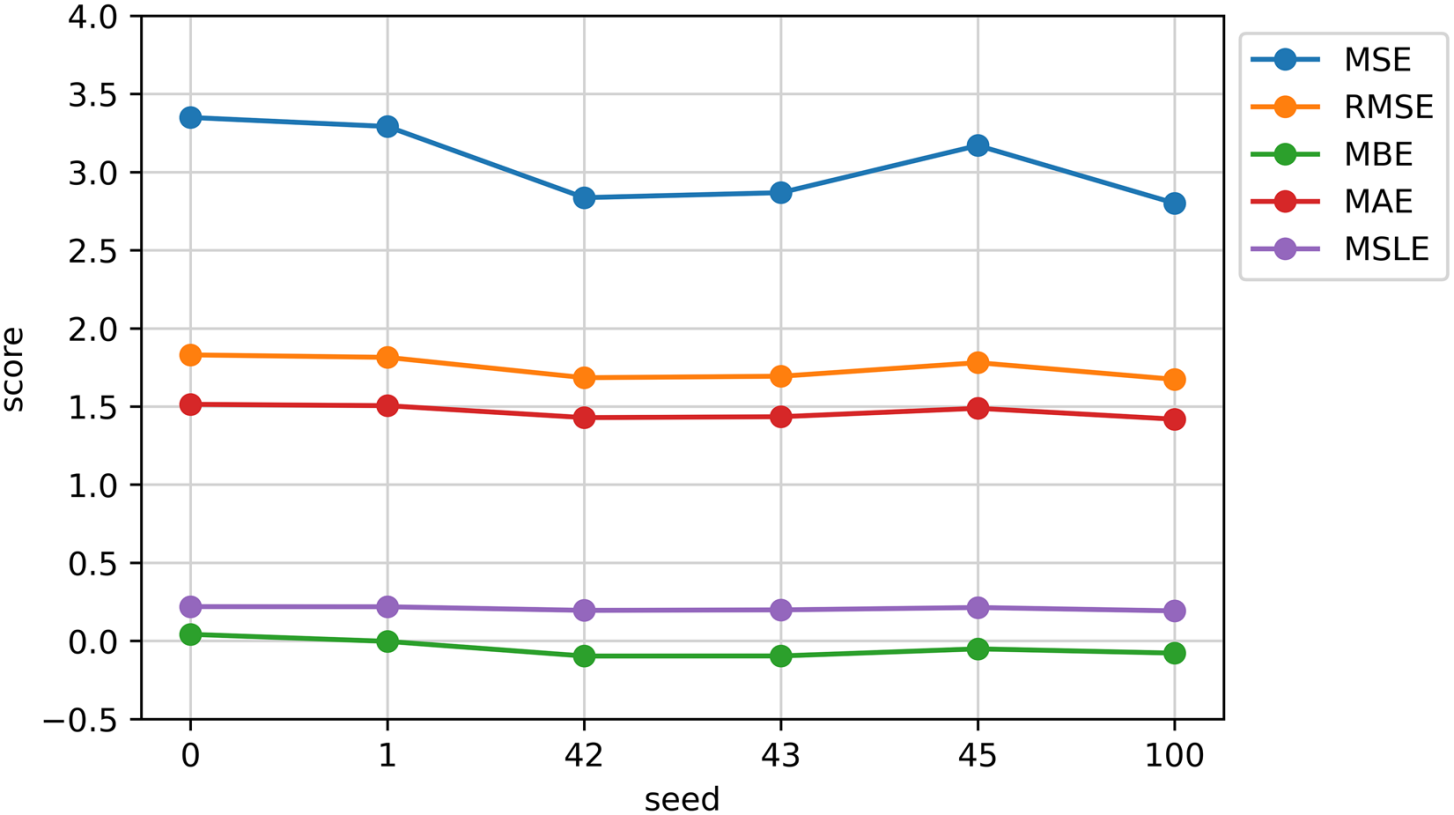
NucleoNetV3 testing results under different seeds.

The use of seed = 43 is to let this experiment reproducible. However, Fig. 6 depicts the testing results from NucleoNetV3 under different seeds but in the same hyperparameters setting. Since the deep neural network follows the stochastic process while training, it is prevalent to get a slightly different result for different seeds.

## Discussions

### Comparison with GGDPR

We presented the performance comparison between the GGDPR model, polygenic OLS regression models, and deep polygenic NucleoNet models, as shown in Table 3. In the OLS model, ENET brought a notable improvement where the MSE score was reduced by 38.67%. However, in NucleoNets, each configuration brought a slight decline in MSE score. With additional modified ENET, the performance of NucleoNetV2 was reduced by 0.07% compared to NucleoNetV1. With additional entropy, the performance of NucleoNetV3 was reduced by 2.24% compared to NucleoNetV2. Nevertheless, the NucleoNets performances resulted in more varied and more numbers of important SNP in exchange. As we can scrutinize in Table 3, the best of NucleoNets, i.e., NucleoNetV1, has an MSE score close to the OLS + ENET model. The NucleoNetV1 reduced an MSE score by 32.28% compared to the basic OLS model.

Let the NucleoNet $\bar{\alpha ^{\prime}}$ stands for an average attention score emerged from 104 testing samples. We found two same important SNPs as the previous research^6^, namely TBGI272457 (NucleoNetV1/ABST-7, GGDP *β* = N/A, OLS $p$-value = 0.728, OLS β = − 0.025, $\bar{\alpha ^{\prime}}$=0.319) and id4009920 (NucleoNetV2/ABST-7, GGDP β = − 0.265, OLS $p$-value = 0.952, OLS *β* = − 0.003, $\bar{\alpha ^{\prime}}$=0.407). The former resided on rice chromosome 6 and position 2,991,002, while the latter resided on rice chromosome 4 and position 30,174,569. id4009920 is a seed-specific protein Bn15D1B^64,65^. TBGI272457 acts as a transporter for anthocyanins vacuolar uptake in rice^66^. Anthocyanins, as members of flavonoid groups, play a role in reproduction and growth, and offer a protection mechanism against biotic or abiotic stress and plaques^67,68^. TBGI272457 is also classified as the NB-ARC domain-containing protein^69^, or resistance proteins (R) which are involved in pathogen recognition and activation of fundamental and innate plant immune system^70,71^. The presence of these genes brings disease resistance capabilities in rice^72^ and hence supports the sustainability of rice yields.

### Indonesian rice yield-associated genes

**Figure 7:**
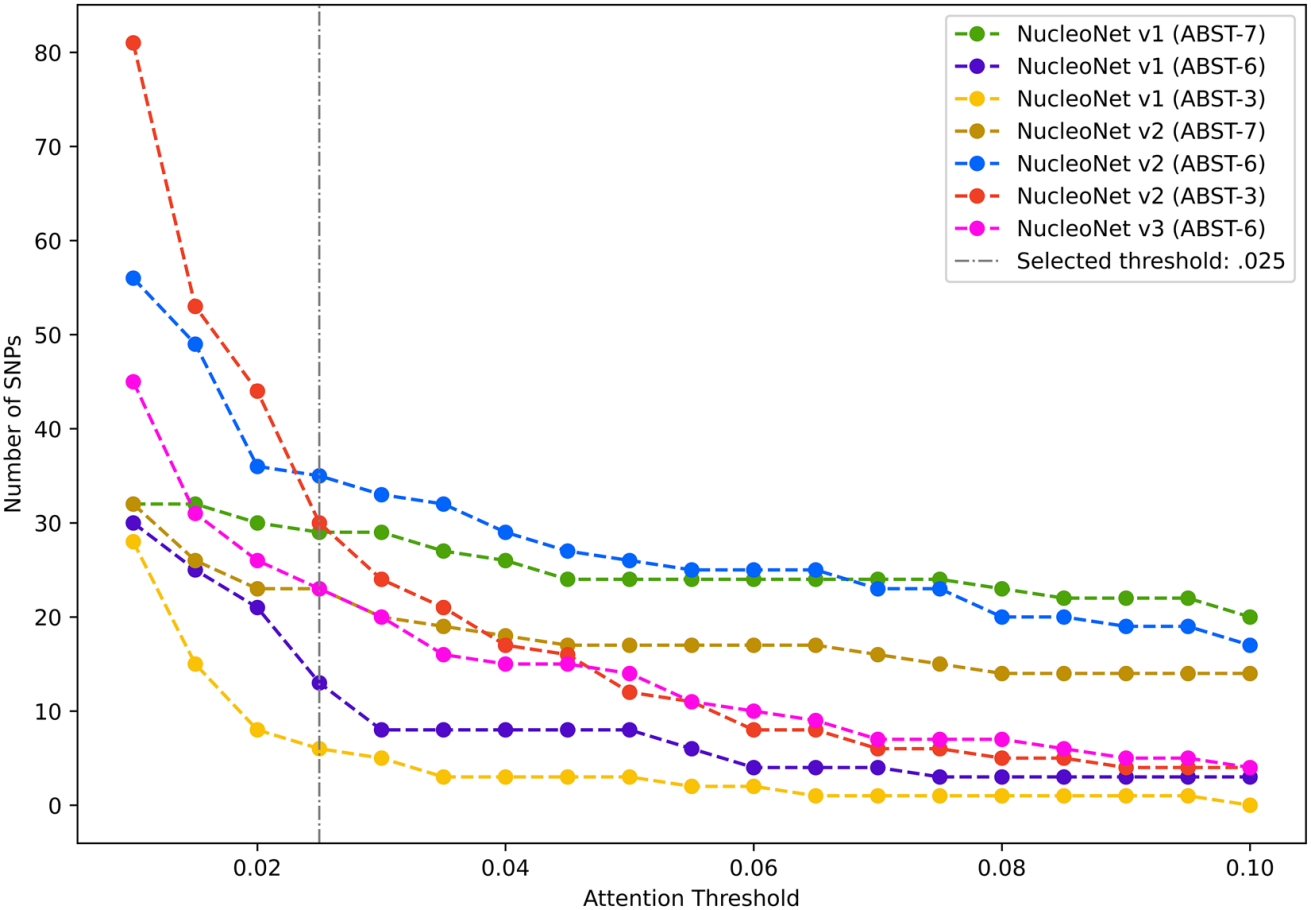
Important SNPs emitted per attention score.

To the day this research is written, there is no prior use of attention score as a fundamental threshold to select important SNPs like $p$-value usually did in GWAS. Therefore, we conducted trials with $\left\{0.01, 0.015,\mathrm{ 0.02,0.025},\dots ,0.1\right\}\in a {^{\prime}}$ in all NucleoNets to see numbers of SNP revealed for each $a {^{\prime}}$. Based on the results presented in Fig. 7, we decided to pick ${a}^{{^{\prime}}}=0.025$ as an ideal and stable threshold since the value beyond it runs into stagnancies and the value behind it provides too diverse numbers of SNP for each NucleoNet model.

Based on this threshold, we summarized the top five important SNPs found by each NucleoNet model, as shown in Table 4. Some of their roles in rice plants were identified and discussed in many studies. For instance, TBGI133263 has a role in rice drought tolerance and photosynthesis mechanism^73^. Its existence was also proved to protect rice seed germination^74^. Its enzyme product, β-Glucosidase, has an impact on the rice root^75,76^. TBGI272488 was discovered as a rice yield-associated gene^77^. The SNP also controls the ATP-binding cassette (ABC) transporters^78–80^ which contributes to multidrug resistance in plants, including rice^81,82^. TBGI336599 was reported to have an impact on rice growth^83^. TBGI130922 controls the metabolism, including the cytokinin metabolism^75^, to support rice coleoptile growth^84^. One product of this gene is flavonoid-biosynthesis networks^85,86^. These flavonoid compounds have many roles in plants, including the reproduction process^87^ and specialized metabolite pathways^88^ in rice. The rest of the SNPs have no further description since they have not been mapped in the rice DNA strand. The other reason is their protein products are still hypothetical. Please refer to Tables 10 and 11 in the Supplementary Information to learn more about these SNPs with their respective genetic details.

**Table 4:** Important SNPs found in the NucleoNets. Chr:Pos means Chromosome:Position. Suffix in each SNP denotes its alternate allele. *Intronic. **Intergenic.

Model	SNP name	Chr:Pos	NucleoNets		Marginal regression		Full regression	
			Count	$\bar{\alpha ^{\prime}}$	$p$-value	$\beta$	$p$-value	$\beta$
NucleoNetV1	TBGI336584_T*	7:28,902,549	104	0.349702	0.692976	0.367086	0.613405	− 0.00464
	TBGI139174_C*	3:10,546,292	100	0.078781	0.501128	0.118872	0.258786	− 0.05250
	TBGI043687_A*	1:27,033,613	98	0.039402	0.461979	0.092519	0.749955	0.018242
	TBGI047097_A*	1:29,101,182	87	0.043968	0.245114	0.146880	0.731616	− 0.00822
	id2008820_T*	2:23,034,401	48	0.028928	0.293053	0.133663	0.487864	− 0.15724
NucleoNetV2	id4010708_C	4:31,871,929	76	0.334360	0.023139	0.178155	0.181538	0.092289
	TBGI133654_T*	3:6,221,117	71	0.073753	0.981030	− 0.00224	0.051080	− 0.11139
	TBGI133263_A**	3:5,884,040	64	0.057674	0.554272	0.060059	0.616267	0.035691
	id1010403_T*	1:16,716,706	53	0.040871	0.275980	0.377068	0.725071	0.007040
	TBGI272488_T*	6:3,001,902	34	0.363929	0.451712	0.057712	0.725524	0.014053
NucleoNetV3	id10004275_C	10:16,252,942	102	0.050838	0.523674	− 0.37561	0.373641	0.050556
	TBGI264076_A*	5:27,953,016	91	0.125639	0.90349	0.018688	0.611320	− 0.01367
	TBGI130922_G**	3:4,441,747	75	0.032907	0.356457	− 0.07551	0.933317	− 0.00536
	TBGI038001_C*	1:23,689,014	73	0.133440	0.564393	− 0.04618	0.195798	− 0.06157
	TBGI336599_C*	7:28,905,733	73	0.043163	0.930258	− 0.00685	0.535020	− 0.03080

### The null hypothesis significance testing

The hypothesis testing (known as NHST) was performed using 38 out of 104 testing data, and thus the degree of freedom is 37. The rest data were excluded due to data distinctions at the time of shuffling the test data for OLS and NucleoNet models. The population to be tested is squared error results from NucleoNetV1 (${\mu }_{V1}$= 2.679, ${\sigma }_{V1}^{2}$ = 7.886), NucleoNetV2 (${\mu }_{V2}$ = 2.642, ${\sigma }_{V2}^{2}$ = 8.166), NucleoNetV3 (${\mu }_{V3}$ = 2.818, ${\sigma }_{V3}^{2}$ = 8.184), OLS (${\mu }_{OLS}$ = 4.758, ${\sigma }_{OLS}^{2}$ = 29.383), and OLS + ENET (${\mu }_{OLS+ENET}$ = 3.121, ${\sigma }_{OLS+ENET}^{2}$ = 8.166). See the full data description in Tables 12, 13, and 14 in the Supplementary Information.

The hypothesis to be tested is as follows. First, for each NucleoNet model $i$, a two-tailed t-test (significance level, ${\alpha }_{sl}=0.025$) is performed to check whether there is a non-zero mean squared error $\mu$ difference compared to the OLS and OLS + ENET models. Statistically, the hypothesis to be tested (two-tailed) between NucleoNets and OLS is defined as ${H}_{0}$: ${\mu }_{i}={\mu }_{OLS}$, ${H}_{1}$: ${\mu }_{i}\ne {\mu }_{OLS}$, while the hypothesis to be tested (two-tailed) between NucleoNets and OLS + ENET is defined as ${H}_{0}$: ${\mu }_{i}={\mu }_{OLS+ENET}$, ${H}_{1}$: ${\mu }_{i}\ne {\mu }_{OLS+ENET}$. The decision rule, if |t-stat|> t-table or $p$-value < ${\alpha }_{sl}$, then we should reject ${H}_{0}$ and proceed to the one-tailed t-test for further investigation.

In a one-tailed t-test scenario (significance level, ${\alpha }_{sl}=0.05$), we checked whether the mean squared error from each NucleoNet model $i$ is less than or greater than the mean squared error from the OLS and OLS + ENET models. Statistically, the hypothesis to be tested (lower one-tailed) between NucleoNets and OLS is defined as ${H}_{0}$: ${\mu }_{i}\nless {\mu }_{OLS}$, ${H}_{1}$: ${\mu }_{i}<{\mu }_{OLS}$, while the hypothesis to be tested (lower one-tailed) between NucleoNets and OLS + ENET is defined as ${H}_{0}$: ${\mu }_{i}\nless {\mu }_{OLS+ENET}$, ${H}_{1}$: ${\mu }_{i}<{\mu }_{OLS+ENET}$. On the contrary, the hypothesis to be tested (upper one-tailed) between NucleoNets and OLS is defined as ${H}_{0}$: ${\mu }_{i}\ngtr {\mu }_{OLS}$, ${H}_{1}$: ${\mu }_{i}>{\mu }_{OLS}$, while the hypothesis to be tested (upper one-tailed) between NucleoNets and OLS + ENET is defined as ${H}_{0}$: ${\mu }_{i}\ngtr {\mu }_{OLS+ENET}$, ${H}_{1}$: ${\mu }_{i}>{\mu }_{OLS+ENET}$. The decision rule for lower one-tailed t-test, if |t-stat|< t-table and $p$-value < ${\alpha }_{sl}$, then we should reject ${H}_{0}$. Meanwhile, the decision rule for upper one-tailed t-test, if |t-stat|> t-table and $p$-value < ${\alpha }_{sl}$, then we should reject ${H}_{0}$. By these settings, NHST results are parsed down in Table 5.

**Table 5:** The NHST results.

Main model	Comparison model	t-test		Validation	Conclusion	Description
NucleoNetV1	OLS	Two-tailed			Reject ${H}_{0}$, accept ${H}_{1}$	Proceed to a one-tailed t-test
		1. |t-stat|> t-table	Is |− 2.998|> 2.026?	TRUE		
		2. $p$-value < ${\alpha }_{sl}$	Is 0.003 < 0.025?	TRUE		
		One-tailed (less than)			Reject ${H}_{0}$, accept ${H}_{1}$	The Indonesian rice yields prediction performance of the NucleoNetV1 model outperformed the OLS model
		1. t-stat < t-table	Is − 2.998 < − 1.687?	TRUE		
		2. $p$-value < ${\alpha }_{sl}$	Is 0.002 < 0.05?	TRUE		
		One-tailed (greater than)			Reject ${H}_{1}$, accept ${H}_{0}$	
		1. t-stat > t-table	Is − 2.998 > 1.687?	FALSE		
		2. $p$-value < ${\alpha }_{sl}$	Is 0.998 < 0.05?	FALSE		
	OLS + ENET	Two-tailed			Reject ${H}_{1}$, accept ${H}_{0}$	The Indonesian rice yields prediction performance of the NucleoNetV1 model has no difference from the OLS + ENET model
		1. |t-stat|> t-table	Is |− 1.028|> 2.026?	FALSE		
		2. $p$-value < ${\alpha }_{sl}$	Is 0.311 < 0.025?	FALSE		
		One-tailed (less than)			–	
		–	–	–		
		One-tailed (greater than)			–	
		–	–	–		
NucleoNetV2	OLS	Two-tailed			Reject ${H}_{0}$, accept ${H}_{1}$	Proceed to a one-tailed t-test
		1. |t-stat|> t-table	Is |− 2.753|> 2.026?	TRUE		
		2. $p$-value < ${\alpha }_{sl}$	Is 0.091 < 0.025?	FALSE		
		One-tailed (less than)			Reject ${H}_{0}$, accept ${H}_{1}$	The Indonesian rice yields prediction performance of the NucleoNetV2 model outperformed the OLS model
		1. t-stat < t-table	Is − 2.753 < − 1.687?	TRUE		
		2. $p$-value < ${\alpha }_{sl}$	Is 0.005 < 0.05?	TRUE		
		One-tailed (greater than)			Reject ${H}_{1}$, accept ${H}_{0}$	
		1. t-stat > t-table	Is − 2.753 > 1.687?	FALSE		
		2. $p$-value < ${\alpha }_{sl}$	Is 0.995 < 0.05?	FALSE		
	OLS + ENET	Two-tailed			Reject ${H}_{1}$, accept ${H}_{0}$	The Indonesian rice yields prediction performance of the NucleoNetV2 model has no difference from the OLS + ENET model
		1. |t-stat|> t-table	Is |− 1.027|> 2.026?	FALSE		
		2. $p$-value < ${\alpha }_{sl}$	Is 0.311 < 0.025?	FALSE		
		One-tailed (less than)			–	
		–	–	–		
		One-tailed (greater than)			–	
		–	–	–		
NucleoNetV3	OLS	Two-tailed			Reject ${H}_{0}$, accept ${H}_{1}$	Proceed to a one-tailed t-test
		1. |t-stat|> t-table	Is |− 2.937|> 2.026?	TRUE		
		2. $p$-value < ${\alpha }_{sl}$	Is 0.006 < 0.025?	TRUE		
		One-tailed (less than)			Reject ${H}_{0}$, accept ${H}_{1}$	The Indonesian rice yields prediction performance of the NucleoNetV3 model outperformed the OLS model
		1. t-stat < t-table	Is − 2.937 < − 1.687?	TRUE		
		2. $p$-value < ${\alpha }_{sl}$	Is 0.003 < 0.05?	TRUE		
		One-tailed (greater than)			Reject ${H}_{1}$, accept ${H}_{0}$	
		1. t-stat > t-table	Is − 2.937 > 1.687?	FALSE		
		2. $p$-value < ${\alpha }_{sl}$	Is 0.997 < 0.05?	FALSE		
	OLS + ENET	Two-tailed			Reject ${H}_{1}$, accept ${H}_{0}$	The Indonesian rice yields prediction performance of the NucleoNetV3 model has no difference from the OLS + ENET model
		1. t-stat < t-table	Is |− 0.743|> 2.026?	FALSE		
		2. $p$-value < ${\alpha }_{sl}$	Is 0.462 < 0.025?	FALSE		
		One-tailed (less than)			–	
		–	–	–		
		One-tailed (greater than)			–	
		–	–	-		

## Conclusions

In this study, a novel deep polygenic neural network named the NucleoNet model was constructed to accurately predict and identify important yield-associated SNPs in Indonesian rice accessions while controlling two major covariates, i.e., location and variety of the samples. The main results and findings are recapitulated as follows: (1) The Indonesian rice yields prediction performance of NucleoNetV1, NucleoNetV2, and NucleoNetV3 outperformed the OLS model. (2) The Indonesian rice yields prediction performance of NucleoNetV1, NucleoNetV2, and NucleoNetV3 has no difference with the OLS + ENET model. (3) Additional entropy penalty in the NucleoNet model brought a more diverse distribution of attention score across SNPs, at the expense of prediction accuracy as a cost. (4) Ablation study showed that the combination of Xavier distribution for weights initialization and Normal distribution for biases initialization sparked more various important SNPs. (5) Two significant SNPs discovered in the prior research, TBGI272457 and id4009920, were also discovered using the NucleoNets.

Since this research is still in its early stages, our future works in the Indonesian rice genomics field will focus on the following things: (1) Extend the covariates, including the influence of pests, pesticides, and climatic information in the year where the rice was planted. (2) Develop a particular deep learning model to impute missing SNPs. (3) Try various attention mechanisms such as self-attention or multi-head attention to improve the SNP significance measurement. (4) Implement the Deep Learning Important Features (DeepLIFT) model to handle SNP significance. (5) Reinforce the deep learning model by instilling it with a novel inductive bias for genomics data. (6) Compare deep learning results with broader common GWAS methods such as LASSO or Bayesian approaches. (7) Develop a biological-based method to validate that important SNPs found in the NucleoNets are useful to increase the annual rice production rate.

## Supplementary Information

Supplementary Information.

## Data Availability

All codes for this research are available at www.github.com/NicholasDominic/The-NucleoNets.
