# Supplementary material for: Deep polygenic neural network for predicting and identifying yield-associated genes in Indonesian rice accessions
Source: Sci Rep. 2022 Aug 15;12:13823. doi: 10.1038/s41598-022-16075-9 (PMC9378700; doi:10.1038/s41598-022-16075-9)
Supplement: Supplementary file 1 — Supplementary Information. [file 41598_2022_16075_MOESM1_ESM.docx]

**A Supplementary Information: Deep polygenic neural network for predicting and identifying yield-associated genes in Indonesian rice accessions**

**Nicholas Dominic**^1*^**, Tjeng Wawan Cenggoro**^2,3^**, Arif Budiarto**^2,3^ **& Bens Pardamean**^1,3^

^1^ Computer Science Department, BINUS Graduate Program - Master of Computer Science Program, Bina Nusantara University, Jakarta 11480, Indonesia

^2^ Computer Science Department, School of Computer Science, Bina Nusantara University, Jakarta 11480, Indonesia

^3^ Bioinformatics and Data Science Research Center, Bina Nusantara University, Jakarta 11480, Indonesia

* Corresponding author: nicholas.dominic@binus.ac.id

Introduction

No supplementary information.

Methods

Data collections.

The raw Indonesian rice genomics data is fully accessible through [BB Biogen website](https://genom.litbang.pertanian.go.id/old_pgpi/pangan/). To see all SNPs, please select the SNP menu and go to “Padi (Indica-Tropical Japonica)”, “Padi (Japonica-Tropical Japonica)” or “Padi (Tropical Japonica)”. The phenotype file describes 12 available rice traits. All of their descriptions is presented in the Table 1.

**Table 1.** Indonesian rice traits

| Rice Trait | Unit | Description |
| --- | --- | --- |
| Days to flowering | - | Days when 50% of the plants have flowers |
| Days to harvest | - | Days until physiological maturity |
| Total tiller | - | Number of tillers per hill |
| Productive tiller | - | Number of tillers that produce panicles |
| Plant height | *cm* | Measured from the ground to the base of the panicle, at the time of flowering |
| Total panicle | - | Total panicles in a square meter |
| Panicle length | *cm* | Main stem panicle length, measured from the base to the tip of the panicle, at 7 days after anthesis (flowers are fully functional) |
| Filled grain | - | The average number of filled grain clumps per panicle |
| Unfilled grain | - | The average number of empty grain clumps per panicle |
| Grain per panicle | - | Total number of grains per panicle |
| KGW | *gram* | Weight of 1000 full grain |
| Yield | *ton/ha* | Tons of rice per hectare |

Genotyping and imputation.

The alternative imputation services that can be used are [Online Plant-ImputeDB](http://gong_lab.hzau.edu.cn/Plant_imputeDB/#!/imputation_rice) or Rice Imputation Server^1^.

SNP validation.

We tried to validate the genotype data with the 18 Million Rice Genome Project (RGP) to see how many Indonesian rice SNPs are overlapped with the global rice data. To achieve this, the raw SNP data in the PLINK format file is used. Before validation, three PLINK files (.bim, .fam, and .bed) should be merged and converted to Variant Call Format (VCF) file by running this command within the PLINK (version 1.9) terminal:

plink --bfile <file_name> --recode vcf

Note that (1) this command is only working in PLINK version 1.9, (2) all three files must be in the same name, and (3) no need to include file extensions when executing the command. The VCF file was then loaded with the Scikit-allele library and rewrote in DataFrame with Pandas library. Now the 18 Million RGP can be easily downloaded from the International Rice Research Institute (IRRI) website. IRRI provides an option ranging from 404,000 to 18 Million RGP data. We selected the highest, i.e., 18,128,777 RGP data, to be precise. Three PLINK files were acquired when downloaded from the given link. The size of BED, BIM, and FAM files are 13.38 Gigabytes, 471.37 Megabytes, and 104 Kilobytes, respectively. To amplify the validation, the paired Chromosome:Position (in String data type) was used as a key. After the validation was completed, it was found that 57 SNPs overlap, as completely inscribed in Table 2.

**Table 2.** Indonesian Rice SNPs Validation with 18 Million RGP Data

| **No.** | **Chromosome** | **Position** | **Reference Allele** | **Alternative Allele** |
| --- | --- | --- | --- | --- |
| 1 | 1 | 5,070,146 | A | G |
| 2 | 1 | 19,918,097 | C | G |
| 3 | 1 | 22,403,832 | T | C |
| 4 | 1 | 23,669,816 | C | A |
| 5 | 1 | 24,259,512 | A | T |
| 6 | 1 | 24,775,992 | T | C |
| 7 | 1 | 24,781,636 | A | C |
| 8 | 1 | 24,850,835 | G | A |
| 9 | 1 | 25,367,449 | A | C |
| 10 | 1 | 27,055,252 | A | G |
| 11 | 1 | 27,414,891 | T | C |
| 12 | 1 | 27,951,045 | C | T |
| 13 | 1 | 28,811,764 | A | G |
| 14 | 1 | 29,062,744 | T | C |
| 15 | 1 | 29,313,930 | T | G |
| 16 | 1 | 29,775,377 | A | G |
| 17 | 1 | 29,839,335 | G | T |
| 18 | 1 | 30,300,392 | A | G |
| 19 | 1 | 30,470,599 | G | T |
| 20 | 1 | 30,736,501 | T | C |
| 21 | 1 | 31907,642 | T | C |
| 22 | 1 | 40,585,518 | A | C |
| 23 | 2 | 201,495 | T | A |
| 24 | 2 | 316,859 | C | T |
| 25 | 2 | 568,585 | G | A |
| 26 | 2 | 786,788 | A | G |
| 27 | 2 | 1,000,481 | G | T |
| 28 | 2 | 2,135,390 | G | A |

**Table 2.** Indonesian Rice SNPs Validation with 18 Million RGP Data (continued)

| **No.** | **Chromosome** | **Position** | **Reference Allele** | **Alternative Allele** |
| --- | --- | --- | --- | --- |
| 29 | 2 | 17,507,106 | G | A |
| 30 | 2 | 23,034,401 | C | T |
| 31 | 2 | 33,146,140 | T | G |
| 32 | 2 | 34,415,952 | T | C |
| 33 | 3 | 4,269,797 | T | C |
| 34 | 3 | 4,487,854 | C | G |
| 35 | 3 | 7,145,866 | C | T |
| 36 | 3 | 10,420,816 | C | T |
| 37 | 3 | 16,819,501 | A | C |
| 38 | 4 | 5,930,532 | C | T |
| 39 | 4 | 18,499,413 | T | C |
| 40 | 5 | 1,221,698 | T | A |
| 41 | 5 | 25,101,446 | A | G |
| 42 | 5 | 29,579,363 | T | G |
| 43 | 6 | 815,178 | A | T |
| 44 | 6 | 4,612,802 | T | C |
| 45 | 7 | 1,899,740 | T | C |
| 46 | 7 | 11,468,087 | G | C |
| 47 | 7 | 27,467,840 | A | T |
| 48 | 7 | 28,290,385 | G | A |
| 49 | 7 | 28,890,105 | G | A |
| 50 | 8 | 3,612,331 | A | G |
| 51 | 9 | 19,067,066 | T | C |
| 52 | 10 | 5,171,461 | A | G |
| 53 | 10 | 15,305,001 | C | G |
| 54 | 10 | 16,252,942 | A | G |
| 55 | 11 | 1,473,671 | T | C |
| 56 | 11 | 21,462,009 | C | G |
| 57 | 12 | 2,341,379 | A | T |

Data preprocessing.

The genetic similarities between each Indonesian rice SNP is depicted in the Figure 1. We measured the skewness and peaks of the rice yield distribution from each location to get a better dataset description. Fisher-Pearson’s Skewness coefficient measures the symmetry of the probability distribution curve. The distribution is positively skewed if its tail extends towards the higher number (to the right), and vice versa. The Skewness coefficient can be obtained by using Equation (1). The perfect skewness has a coefficient of 0. However, the Fisher’s Kurtosis coefficient measures the flatness of the probability distribution curve. The Kurtosis coefficient can be obtained by using Equation (2). The perfect Fisher’s Kurtosis coefficient is 0 or called the mesokurtic. The high peak in the distribution curve ($\gamma_{2}>0$) is called leptokurtic, while the low peak ($\gamma_{2}<0$) is called platykurtic. From the equations, $\gamma_{1}$ means the Fisher-Pearson’s Skewness coefficient and $\gamma_{2}$ means the Fisher’s Kurtosis coefficient. $n$ denotes the total sample, $x_{i}$ denotes the data point, $\bar{x}$ denotes the mean of all samples, and $\sigma$ means the dataset standard deviation.

|  | $\gamma_{1}=\frac{\frac{1}{n}\sum_{i=1}^{n} \left( x_{i}-\bar{x} \right)^{3}}{\sigma^{3}}$ |  |
| --- | --- | --- |

|  | $\gamma_{2}=\frac{\frac{1}{n}\sum_{i=1}^{n} \left( x_{i}-\bar{x} \right)^{4}}{\sigma^{4}}-3$ |  |
| --- | --- | --- |

When we processed the genotype dataset, all SNPs should be encoded. How these inputs are encoded affects the machine learning algorithm performance. Unlike DNA encodings^2–6^, SNP can be encoded as an additive model, recessive/dominant model, one-hot vector^7^, hexadecimal format, and graphical 1D/2D linear format^8^. In the additive model, all SNP alleles are encoded according to the total of its alternative allele (Alt) as it represents a mutation in one locus. For clarity, notice the example shown in Table 3. This encoding type was selected because it has been proven to have a higher Area Under ROC Curve (AUC) value compared to the one-hot or recessive/dominant model^9^.


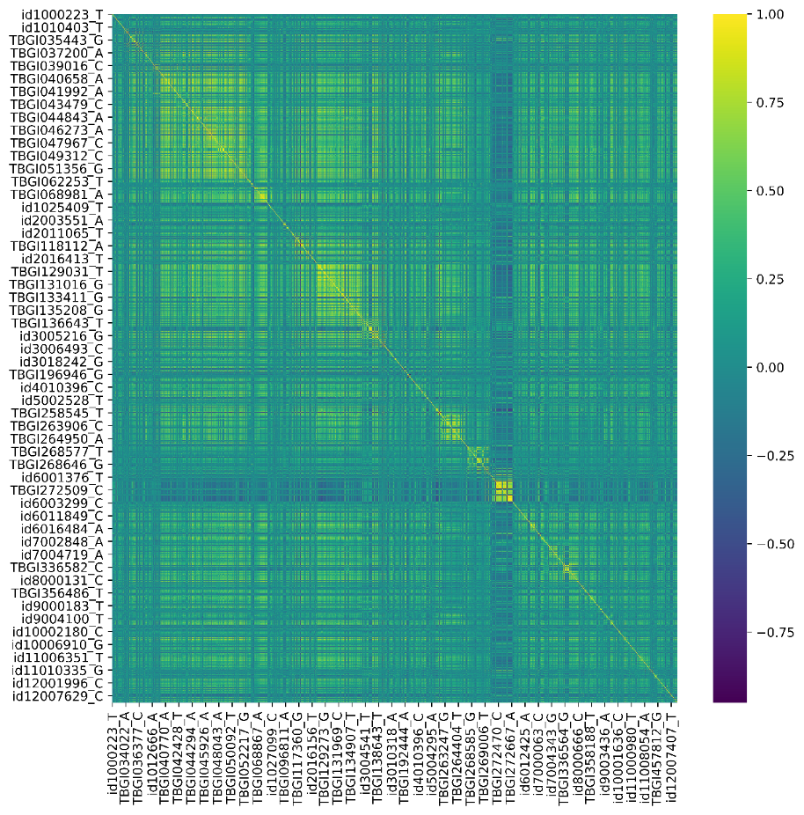


**Figure 1.** Genetic similarities between each Indonesian rice SNP

**Table 3.** SNP encodings based on the alternate alleles

| ID | [Ref/Alt] Allele | SNP Allele 1 | SNP Allele 2 | Encoding | SNP Type |
| --- | --- | --- | --- | --- | --- |
| rs01 | [A/**T**] | A | A | **0** | Homozygous major/reference |
| rs02 | [G/**C**] | G | **C** | **1** | Heterozygous |
| rs03 | [C/**T**] | **T** | C | **1** | Heterozygous |
| rs04 | [A/**G**] | **G** | **G** | **2** | Homozygous minor/alternate |

Regression modeling.

No supplementary information.

The NucleoNet modeling.

No supplementary information.

Ablation study.

See Table 4 for each ablation study configuration.

Evaluation metrics.

Even it seems a trivial issue, we have a strong reason for the selection of our six prediction accuracy measures. First, MSE measures a variance of residuals, while RMSE measures a standard deviation of residuals. MSE is sensitive to outliers because the error values will be greatly increased if the error values $e>1$. However, in the deep learning training, MSE performs better and is more stable than any loss function because its gradient (derivative) will decrease towards $e\approx0$, even with the fixed learning rate setting.

On the other hand, MAE measures an average of residuals without considering their direction (absolute), and it is more robust to outliers since $e^{2}>\left| e \right|$. Unlike MSE, MAE is not used in deep learning training because the metric will induce a large gradient even for small errors. The issue may be tackled by using the dynamic learning rate. On the contrary, MBE is the same characteristics as MAE unless it considers the direction.

MSLE tells about the percentual difference, which means it treats the small and big ranges of measured values approximately the same while calculating the error. Unlike MSE that will penalize the underestimate and overestimate results the same, MSLE has an advantage to always penalize more on the underestimated rather than the overestimated value.

Mean Absolute Percentage Error (MAPE) seems to be commonly used as the prediction accuracy measurement. However, some drawbacks were encountered when using MAPE^74–76^. For instance, it only works well on the strict positive data. Another drawback is MAPE has an asymmetrical attribute, which means interchanging predicted and true values within the formula do not lead to the same outcome. Recall that any accuracy measure should fulfill several properties such as symmetry, additivity, continuity, homogeneity, and interpretability^61,77^. This issue was then resolved using the Symmetric Mean Absolute Percentage Error (SMAPE)^78^. Another promising metric is the Log Accuracy Ratio Error $\sum\ln\left( \frac{\hat{y}}{y} \right)^{2}$, but we decided to exclude it since it is more suitable for a multiplicative or heteroscedastic error^79^.

Hardware, software, and libraries.

No supplementary information.

**Table 4.** The ablation studies

|  | ABST-1 | ABST-2 | ABST-3 | ABST-4 | ABST-5 | ABST-6 | ABST-7 |
| --- | --- | --- | --- | --- | --- | --- | --- |
| AT1.$w$ | Default | $w_{a1}\mathcal{\sim U}\left( -g_{l}\sqrt{\frac{1}{f_{i}}},g_{l}\sqrt{\frac{1}{f_{i}}} \right)$ | $w_{a1}\mathcal{\sim N}\left( 0,g_{r}\sqrt{\frac{2}{f_{i}+f_{o}}} \right)$ | $w_{a1}\mathcal{\sim N}\left( 0, 1 \right)$ | $w_{a1}\mathcal{\sim U}\left( 0, 1 \right)$ | $w_{a1}\mathcal{\sim N}\left( 0,g_{r}\sqrt{\frac{2}{f_{i}+f_{o}}} \right)$ | $w_{a1}\mathcal{\sim N}\left( 0, 1 \right)$ |
| AT1.$b$ | Default | $b_{a1}\mathcal{\sim U}\left( -g_{l}\sqrt{\frac{1}{f_{i}}},g_{l}\sqrt{\frac{1}{f_{i}}} \right)$ | $b_{a1}\mathcal{\sim N}\left( 0, 1 \right)$ | $b_{a1}\mathcal{\sim N}\left( 0, 1 \right)$ | $b_{a1}\mathcal{\sim U}\left( 0, 1 \right)$ | $b_{a1}\mathcal{\sim N}\left( 0, 1 \right)$ | $b_{a1}\mathcal{\sim N}\left( 0, 1 \right)$ |
| AT2.$w$ | Default | $w_{a2}\mathcal{\sim U}\left( -g_{l}\sqrt{\frac{1}{f_{i}}},g_{l}\sqrt{\frac{1}{f_{i}}} \right)$ | $w_{a2}\mathcal{\sim N}\left( 0,g_{r}\sqrt{\frac{2}{f_{i}+f_{o}}} \right)$ | $w_{a2}\mathcal{\sim N}\left( 0, 1 \right)$ | $w_{a2}\mathcal{\sim U}\left( 0, 1 \right)$ | $w_{a2}\mathcal{\sim N}\left( 0, 1 \right)$ | $w_{a2}\mathcal{\sim N}\left( 0,g_{r}\sqrt{\frac{2}{f_{i}+f_{o}}} \right)$ |
| AT2.$b$ | Default | $b_{a2}\mathcal{\sim U}\left( -g_{l}\sqrt{\frac{1}{f_{i}}},g_{l}\sqrt{\frac{1}{f_{i}}} \right)$ | $b_{a2}\mathcal{\sim N}\left( 0, 1 \right)$ | $b_{a2}\mathcal{\sim N}\left( 0, 1 \right)$ | $b_{a2}\mathcal{\sim U}\left( 0, 1 \right)$ | $b_{a2}\mathcal{\sim N}\left( 0, 1 \right)$ | $b_{a2}\mathcal{\sim N}\left( 0, 1 \right)$ |
| SD | Default | $w_{s},b_{s}\mathcal{\sim N}\left( 0, 1 \right)$ | $w_{s},b_{s}\mathcal{\sim N}\left( 0, 1 \right)$ | $w_{s},b_{s}\mathcal{\sim N}\left( 0, 1 \right)$ | $w_{s},b_{s}\mathcal{\sim N}\left( 0, 1 \right)$ | $w_{s},b_{s}\mathcal{\sim N}\left( 0, 1 \right)$ | $w_{s},b_{s}\mathcal{\sim N}\left( 0, 1 \right)$ |
| SP | Default | $w_{sp},b_{sp}\mathcal{\sim N}\left( 0, 1 \right)$ | $w_{sp},b_{sp}\mathcal{\sim N}\left( 0, 1 \right)$ | $w_{sp},b_{sp}\mathcal{\sim N}\left( 0, 1 \right)$ | $w_{sp},b_{sp}\mathcal{\sim N}\left( 0, 1 \right)$ | $w_{sp},b_{sp}\mathcal{\sim N}\left( 0, 1 \right)$ | $w_{sp},b_{sp}\mathcal{\sim N}\left( 0, 1 \right)$ |
| SL | Default | $w_{sl},b_{sl}\mathcal{\sim N}\left( 0, 1 \right)$ | $w_{sl},b_{sl}\mathcal{\sim N}\left( 0, 1 \right)$ | $w_{sl},b_{sl}\mathcal{\sim N}\left( 0, 1 \right)$ | $w_{sl},b_{sl}\mathcal{\sim N}\left( 0, 1 \right)$ | $w_{sl},b_{sl}\mathcal{\sim N}\left( 0, 1 \right)$ | $w_{sl},b_{sl}\mathcal{\sim N}\left( 0, 1 \right)$ |
| SV | Default | $w_{sv},b_{sv}\mathcal{\sim N}\left( 0, 1 \right)$ | $w_{sv},b_{sv}\mathcal{\sim N}\left( 0, 1 \right)$ | $w_{sv},b_{sv}\mathcal{\sim N}\left( 0, 1 \right)$ | $w_{sv},b_{sv}\mathcal{\sim N}\left( 0, 1 \right)$ | $w_{sv},b_{sv}\mathcal{\sim N}\left( 0, 1 \right)$ | $w_{sv},b_{sv}\mathcal{\sim N}\left( 0, 1 \right)$ |
| FC | Default | $w_{fc},b_{fc}\mathcal{\sim N}\left( 0, 1 \right)$ | $w_{fc},b_{fc}\mathcal{\sim N}\left( 0, 1 \right)$ | $w_{fc},b_{fc}\mathcal{\sim N}\left( 0, 1 \right)$ | $w_{fc},b_{fc}\mathcal{\sim N}\left( 0, 1 \right)$ | $w_{fc},b_{fc}\mathcal{\sim N}\left( 0, 1 \right)$ | $w_{fc},b_{fc}\mathcal{\sim N}\left( 0, 1 \right)$ |

**Abbreviations:**

AT1.$w$: Attention Layer 1 (weight), AT1.$b$: Attention Layer 1 (bias), AT2.$w$: Attention Layer 2 (weight), AT2.$b$: Attention Layer 2 (bias), SD: SNP Data Embedding, SP: SNP Position Data Embedding, SL: Sample Location Data Embedding, SV: Sample Variety Data Embedding, FC: Fully Connected Layer in Deep Model.

Results

Statistical analysis.

**Table 5.** Indonesian rice yield data descriptions

| Location | Total Sample | | | Mean  ± Std | Skewness Coef. | | Kurtosis Coef. | | Rice Yield  Distribution Histogram |
| --- | --- | --- | --- | --- | --- | --- | --- | --- | --- |
| All | 687 | | | 3.44  ± 1.85 | 0.53 | | -0.06 | | 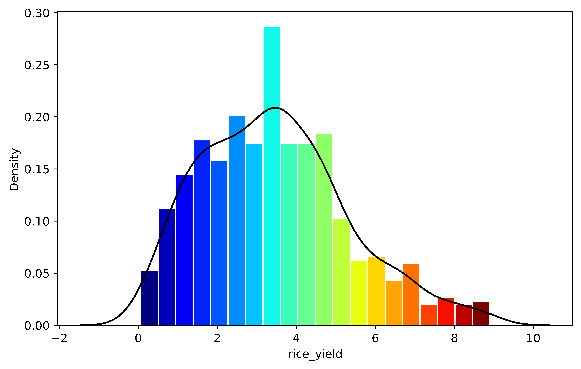 |
| Kuningan | 150 | | | 5.01  ± 1.98 | 0.14 | | -0.86 | | 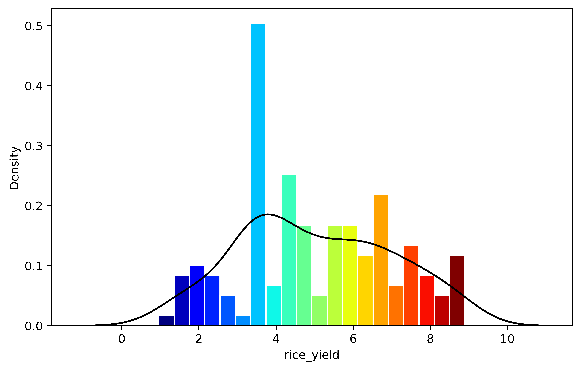 |
| Subang | | 124 | 3.62  ± 1.82 | | | 0.08 | | -0.85 | 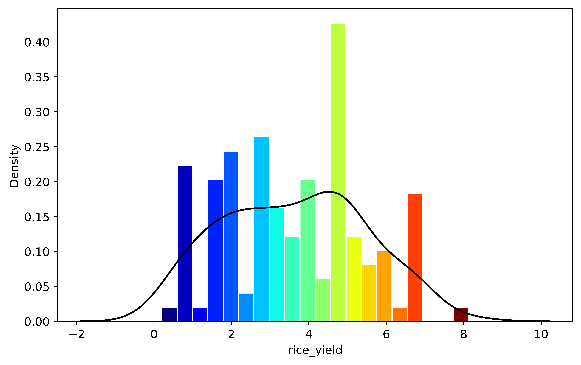 |
| Citayam | | 413 | 2.83  ± 1.43 | | | 0.19 | | -0.61 | 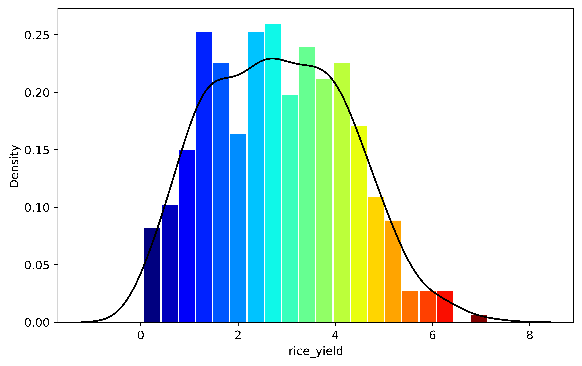 |

Ordinary Least Squares results.

**Table 6.** Full OLS regression for significant SNPs

| Measures | Experiment 1 | Experiment 2 | Prior Research |
| --- | --- | --- | --- |
| Total data | 16 | 36 | 9 |
| MSE | 3.076 | 3.089 | 3.351 |
| RMSE | 1.754 | 1.758 | 1.831 |
| MBE | -0.297 | -0.351 | -0.407 |
| MAE | 1.473 | 1.486 | 1.532 |
| MSLE | 0.223 | 0.223 | 0.244 |
| SMAPE | 50.021% | 50.108% | 51.211% |

**Table 7.** Full OLS with Elastic Net for significant SNPs

| Measures | Experiment 1 | Experiment 2 | Prior Research |
| --- | --- | --- | --- |
| Total data | 16 | 36 | 9 |
| MSE | 2.467 | 2.634 | 2.445 |
| RMSE | 1.571 | 1.623 | 1.564 |
| MBE | 0.096 | 0.079 | 0.117 |
| MAE | 1.276 | 1.319 | 1.271 |
| MSLE | 0.143 | 0.152 | 0.136 |
| SMAPE | 40.806% | 41.856% | 40.286% |


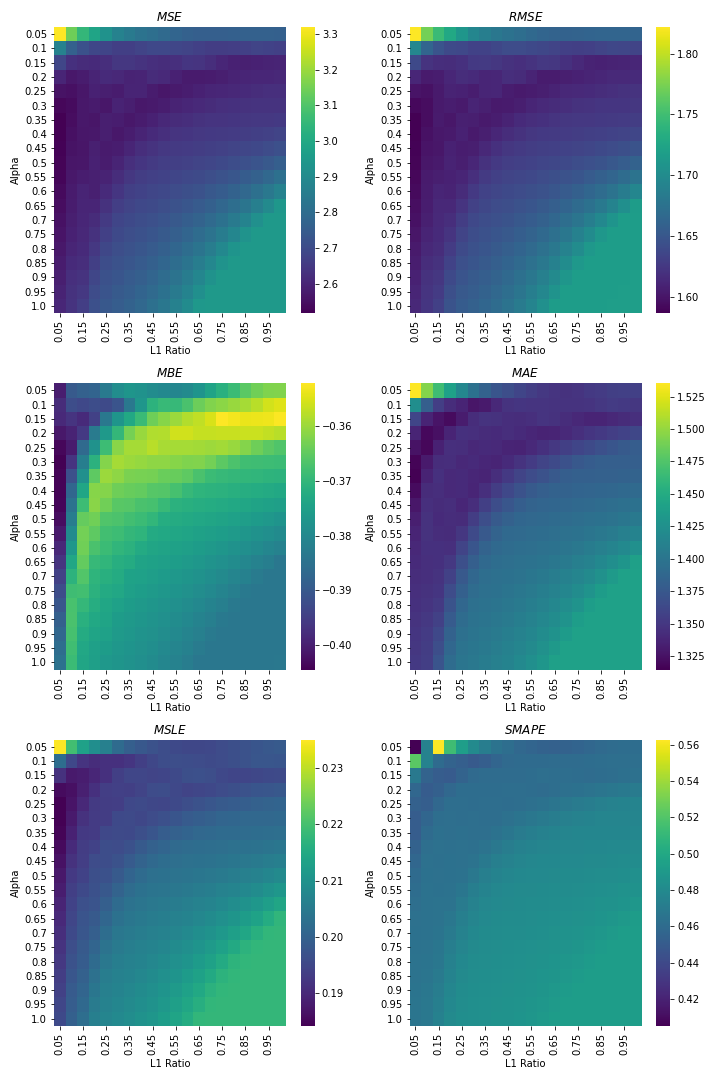


**Figure 2.** The Elastic Net hyperparameter configuration

The NucleoNets results.

**Table 8.** The best hyperparameters for the NucleoNets

| Hyperparameter | Search Space | Exp. 1  Best Value | Exp. 2  Best Value | Exp. 3  Best Value |
| --- | --- | --- | --- | --- |
| Attention hidden layer (*a*_h_) | [10, 20, 30] | 10 | 20 | 10 |
| MLP hidden layer (*m*_h_) | [32, 64, 128, 256] | 32 | 128 | 64 |
| Embedding dimension (*e*) | [16, 32, 64, 128] | 32 | 64 | 32 |
| Batch size (*b*) | [16, 32, 64, 128] | 32 | 128 | 16 |
| Learning rate | [1e-4, 1e-5, 1e-6] | 1e-4 | 1e-4 | 1e-5 |
| Regularization lambda 1 | [0.1, 0.2, 0.3, …, 0.9] | - | 0.8 | 0.8 |
| Regularization lambda 2 | [0.1, 0.2, 0.3, …, 0.9] | - | 0.3 | 0.3 |
| Regularization weight | [1.0, 0.1, 0.01, 0.001, 0.0001] | - | 0.1 | 0.001 |
| Entropy weight | [0.01, 0.02, 0.03, …, 0.09] | - | - | 0.05 |
| Validation MSE |  | 3.032 | 3.097 | 3.233 |
| Execution time (ET) |  | 1h 03m 05s  (390.33s/trial) | 1h 16m 02s  (456.22s/trial) | 1h 35m 56s  (575.63s/trial) |

**Table 9.** The NucleoNets testing results for all ablation studies (ABST)

|  | ABST-1 | ABST-2 | ABST-3 | ABST-4 | ABST-5 | | ABST-6 | ABST-7 |
| --- | --- | --- | --- | --- | --- | --- | --- | --- |
| *Experiment 1 (NucleoNetV1)* | | | | | | | | |
| MSE | 2.890 | 2.843 | 2.785 | 2.813 | N/A | | **2.779** | 2.794 |
| RMSE | 1.700 | 1.686 | 1.669 | 1.677 | N/A | | **1.667** | 1.672 |
| MBE | -0.275 | -0.181 | 0.055 | 0.094 | N/A | | **0.099** | 0.079 |
| MAE | 1.441 | 1.432 | 1.413 | 1.416 | N/A | | **1.407** | 1.413 |
| MSLE | 0.205 | 0.198 | 0.186 | 0.186 | N/A | | **0.184** | 0.184 |
| SMAPE | 47.397% | 47.328% | 47.265% | 47.399% | N/A | | **47.156%** | 47.212% |
| ET | 1,620s | 1,820s | 1,730s | 1,530s | 0s | | **1,630s** | 1,890s |
| *Experiment 2 (NucleoNetV2)* | | | | | | | | |
| MSE | - | - | **2.782** | - | | - | 2.799 | 3.035 |
| RMSE | - | - | **1.668** | - | | - | 1.673 | 1.742 |
| MBE | - | - | **0.084** | - | | - | 0.015 | -0.485 |
| MAE | - | - | **1.412** | - | | - | 1.412 | 1.467 |
| MSLE | - | - | **0.183** | - | | - | 0.191 | 0.218 |
| SMAPE | - | - | **47.548%** | - | | - | 47.960% | 47.359% |
| ET | - | - | **5,890s** | - | | - | 5,120s | 4,280s |
| *Experiment 3 (NucleoNetV3)* | | | | | | | | |
| MSE | - | - | - | - | | - | **2.863** | - |
| RMSE | - | - | - | - | | - | **1.692** | - |
| MBE | - | - | - | - | | - | **-0.074** | - |
| MAE | - | - | - | - | | - | **1.433** | - |
| MSLE | - | - | - | - | | - | **0.197** | - |
| SMAPE | - | - | - | - | | - | **47.481%** | - |
| ET | - | - | - | - | | - | **4,910s** | - |

Ablation study.

PyTorch initializes the neural network weight and bias within the Kaiming Uniform distribution, as reported in the webpage [documentation](https://pytorch.org/docs/stable/generated/torch.nn.Linear.html) or GitHub [codes](https://github.com/pytorch/pytorch/blob/master/torch/nn/modules/linear.py#L96).

Discussions

Comparison with GGDPR.

No supplementary information.

Indonesian rice yield-associated genes.

Details of each detected important SNP, including its ID in different institutions ([Ensembl](https://plants.ensembl.org/Oryza_sativa/Info/Index), Michigan State University/MSU, and National Center for Biotechnology Information/[NCBI](https://www.ncbi.nlm.nih.gov/gene/)) are presented in Table 10 and Table 11.

**Table 10.** Indonesian rice SNP alternative name/ID

| **SNP Name** | **Alternate Allele** | **Ensembl ID** | **MSU ID** | **NCBI ID** |
| --- | --- | --- | --- | --- |
| TBGI336584 | T | Os07g0681600 | LOC_Os07g48360 | [LOC4344306](https://www.ncbi.nlm.nih.gov/gene/?term=Os07g0681600) |
| TBGI139174 | C | Os03g0299900 | LOC_Os03g18810 | [LOC4332563](https://www.ncbi.nlm.nih.gov/gene/?term=LOC_Os03g18810) |
| TBGI043687 | A | - | LOC_Os01g47290 | - |
| TBGI047097 | A | - | LOC_Os01g50670 | - |
| id2008820 | T | - | LOC_Os02g38100 | - |
| id4010708 | C | N/A | N/A | N/A |
| TBGI133654 | T | Os03g0218200 | LOC_Os03g11874 | [LOC9271769](https://www.ncbi.nlm.nih.gov/gene/?term=Os03g0218200) |
| TBGI133263 | A | Os03g0212800 | LOC_Os03g11420 | [LOC4332041](https://www.ncbi.nlm.nih.gov/gene/?term=LOC_Os03g11420) |
| id1010403 | T | Os01g0393000 | LOC_Os01g29830 | - |
| TBGI272488 | T | Os06g0158900 | LOC_Os06g06440 | [LOC4340195](https://www.ncbi.nlm.nih.gov/gene/?term=Os06g0158900) |
| id10004275 | C | N/A | N/A | N/A |
| TBGI264076 | A | Os05g0561800 | LOC_Os05g48770 | - |
| TBGI130922 | G | Os03g0184550 | LOC_Os03g08624 | [LOC9269735](https://www.ncbi.nlm.nih.gov/gene/?term=Os03g0184550) |
| TBGI038001 | C | - | LOC_Os01g41834 | - |
| TBGI336599 | C | Os07g0681600 | LOC_Os07g48360 | [LOC4344306](https://www.ncbi.nlm.nih.gov/gene/?term=Os07g0681600) |

**Table 11.** Indonesian rice SNP genetics details

| **SNP Name** | **Region** | **Gene Range (Chr:Pos)** | **Strand** | GC Content Ratio |
| --- | --- | --- | --- | --- |
| TBGI336584 | Intronic | [7:28,901,514-28,908,685](https://plants.ensembl.org/Oryza_sativa/Location/View?r=7%3A28902549-28903549;site=ensemblunit) | Forward | 39.12 |
| TBGI139174 | Intronic | [3:10,542,880-10,546,261](https://plants.ensembl.org/Oryza_sativa/Location/View?r=3%3A10545292-10546292;site=ensemblunit) | Forward | 42.25 |
| TBGI043687 | Intronic | [1:27,031,779-27,032,719](https://plants.ensembl.org/Oryza_sativa/Location/View?r=1%3A27033613-27034613;site=ensemblunit) | Forward | - |
| TBGI047097 | Intronic | [1:29,100,651-29,101,322](https://plants.ensembl.org/Oryza_sativa/Location/View?r=1%3A29101182-29102182;site=ensemblunit) | Forward | - |
| id2008820 | Intronic | [2:23,034,228-23,035,589](https://plants.ensembl.org/Oryza_sativa/Location/View?r=2%3A23034401-23035401;site=ensemblunit) | Forward | - |
| id4010708 | N/A | N/A | N/A | N/A |
| TBGI133654 | Intronic | [3:6,218,055-6,225,078](https://plants.ensembl.org/Oryza_sativa/Location/View?r=3%3A6221117-6222117;site=ensemblunit) | Reverse | 50.05 |
| TBGI133263 | Intergenic | [3:5,876,410-5,883,222](https://plants.ensembl.org/Oryza_sativa/Location/View?r=3%3A5883040-5884040;site=ensemblunit) | Reverse | 45.71 |
| id1010403 | Intronic | [1:16,716,670-16,719,374](https://plants.ensembl.org/Oryza_sativa/Location/View?r=1%3A16716706-16717706;site=ensemblunit) | Forward | 42.11 |
| TBGI272488 | Intronic | [6:3,001,438-3,009,777](https://plants.ensembl.org/Oryza_sativa/Location/View?r=6%3A3001902-3002902;site=ensemblunit) | Reverse | 38.59 |
| id10004275 | N/A | N/A | N/A | N/A |
| TBGI264076 | Intronic | [5:27,950,781-27,955,317](https://plants.ensembl.org/Oryza_sativa/Location/View?r=5%3A27952016-27953016;site=ensemblunit) | Reverse | 41.28 |
| TBGI130922 | Intergenic | [3:4,441,960-4,443,828](https://plants.ensembl.org/Oryza_sativa/Location/View?r=3%3A4441747-4442747;site=ensemblunit) | Forward | 47.03 |
| TBGI038001 | Intronic | [1:23,688,289-23,692,031](https://plants.ensembl.org/Oryza_sativa/Location/View?r=1%3A23689014-23690014;site=ensemblunit) | Reverse | - |
| TBGI336599 | Intronic | [7:28,901,514-28,908,685](https://plants.ensembl.org/Oryza_sativa/Location/View?r=7%3A28905733-28906733;site=ensemblunit) | Forward | 39.12 |

The Null Hypothesis Significance Testing.

In Table 12, Table 13., and Table 14., the complete t-test data for NucleoNetV1, NucleoNetV2, and NucleoNetV3 models are presented. For your convenience, we format each table as close as possible to the Excel t-test calculation table format.

**Table 12.** t-test Data for NucleoNetV1

| Model | NucleoNetV1 | OLS | | NucleoNetV1 | OLS+ENET |
| --- | --- | --- | --- | --- | --- |
| Number of observations | 38 | 38 | | 38 | 38 |
| Mean of squared error | 2.679 | 4.758 | | 2.679 | 3.121 |
| Variance of squared error | 7.886 | 29.383 | | 7.886 | 8.166 |
| Degree of freedom | 37 | 37 | | 37 | 37 |
| **t-test / t-stat** | **-2.998** | | **-1.028** | | |
| *p*-value lower one-tailed | 0.002 | | 0.155 | | |
| *p*-value upper one-tailed | 0.998 | | 0.844 | | |
| t Critical / t-table one-tailed | 1.687 | | 1.687 | | |
| *p*-value two-tailed | 0.004 | | 0.311 | | |
| t Critical / t-table two-tailed | 2.026 | | 2.026 | | |

**Table 13.** t-test Data for NucleoNetV2

| Model | NucleoNetV2 | OLS | | NucleoNetV2 | OLS+ENET |
| --- | --- | --- | --- | --- | --- |
| Number of observations | 38 | 38 | | 38 | 38 |
| Mean of squared error | 2.642 | 4.758 | | 2.642 | 3.121 |
| Variance of squared error | 8.166 | 29.383 | | 8.166 | 8.166 |
| Degree of freedom | 37 | 37 | | 37 | 37 |
| **t-test / t-stat** | **-2.753** | | **-1.027** | | |
| *p*-value lower one-tailed | 0.005 | | 0.156 | | |
| *p*-value upper one-tailed | 0.995 | | 0.844 | | |
| t Critical / t-table one-tailed | 1.687 | | 1.687 | | |
| *p*-value two-tailed | 0.091 | | 0.311 | | |
| t Critical / t-table two-tailed | 2.026 | | 2.026 | | |

Conclusions

No supplementary information.

References

1. Wang, D. R. *et al.* An imputation platform to enhance integration of rice genetic resources. *Nat. Commun.* **9**, 1–10 (2018).

2. Yu, H. & Dai, Z. SNNRice6mA: A Deep Learning Method for Predicting DNA N6-Methyladenine Sites in Rice Genome. *Front. Genet.* **10**, 1–6 (2019).

3. Zou, J. *et al.* A primer on deep learning in genomics. *Nat. Genet.* **51**, 12–18 (2019).

4. Min, X. *et al.* Predicting enhancers with deep convolutional neural networks. *BMC Bioinformatics* **18**, (2017).

5. Du, J. *et al.* Gene2vec: Distributed representation of genes based on co-expression. *BMC Genomics* **20**, (2019).

6. Nguyen, N. G. *et al.* DNA Sequence Classification by Convolutional Neural Network. *J. Biomed. Sci. Eng.* **09**, 280–286 (2016).

7. Mittag, F., Römer, M. & Zell, A. Influence of feature encoding and choice of classifier on disease risk prediction in genome-wide association studies. *PLoS One* **10**, (2015).

**Table 14.** t-test Data for NucleoNetV3

| Model | NucleoNetV3 | OLS | | NucleoNetV3 | OLS+ENET |
| --- | --- | --- | --- | --- | --- |
| Number of observations | 38 | 38 | | 38 | 38 |
| Mean of squared error | 2.818 | 4.758 | | 2.818 | 3.121 |
| Variance of squared error | 8.184 | 29.383 | | 8.184 | 8.166 |
| Degree of freedom | 37 | 37 | | 37 | 37 |
| **t-test / t-stat** | **-2.937** | | **-0.743** | | |
| *p*-value lower one-tailed | 0.003 | | 0.231 | | |
| *p*-value upper one-tailed | 0.997 | | 0.769 | | |
| t Critical / t-table one-tailed | 1.687 | | 1.687 | | |
| *p*-value two-tailed | 0.006 | | 0.462 | | |
| t Critical / t-table two-tailed | 2.026 | | 2.026 | | |

8. Garafutdinov, R. R., Sakhabutdinova, A. R., Slominsky, P. A., Aminev, F. G. & Chemeris, A. V. A new digital approach to SNP encoding for DNA identification. *Forensic Sci. Int.* **317**, 0–4 (2020).

9. Nababan, E. B., Nurhasanah, R. & Huzaifah, A. S. Web Based Application for Controlling Data Quality in Phenotype Prediction of Indonesian Rice Genomes. *J. Phys. Conf. Ser.* **1566**, (2020).

10. Mcmahan, C. *et al.* A Bayesian hierarchical model for identifying significant polygenic effects while controlling for confounding and repeated measures. *Stat. Appl. Genet. Mol. Biol.* **16**, 407–419 (2017).

11. Bahdanau, D., Cho, K. H. & Bengio, Y. Neural machine translation by jointly learning to align and translate. *3rd Int. Conf. Learn. Represent. ICLR 2015 - Conf. Track Proc.* 1–15 (2015).
